# Gene-expression signature functional annotation of breast cancer tumours in function of age

**DOI:** 10.1186/s12920-015-0153-6

**Published:** 2015-11-23

**Authors:** Pascal Jézéquel, Zein Sharif, Hamza Lasla, Wilfried Gouraud, Catherine Guérin-Charbonnel, Loïc Campion, Stéphane Chrétien, Mario Campone

**Affiliations:** Bioinfomics unit, Integrated Centre for Oncology - René Gauducheau, Bd J. Monod, Nantes, Saint Herblain Cedex 44805 France; Cancer Genomic Unit, Integrated Centre for Oncology - René Gauducheau, Bd J. Monod, Nantes, Saint Herblain Cedex 44805 France; INSERM U892, IRT-UN, 8 quai Moncousu, Nantes Cedex, 44007 France; Départemental de Vendée - site de Montaigu, Polyvalent medicine service, Centre Hospitalier, 54, rue Saint Jacques, BP 259, Montaigu, 85602 France; Biostatistics unit, Integrated Centre for Oncology - René Gauducheau, Bd J. Monod, Nantes, Saint Herblain Cedex 44805 France; Mathematics laboratory, UMR CNRS 6623 et Université de Franche Comté, 16 route de Gray, Besançon Cedex, 25030 France; Medical oncology service, Integrated Centre for Oncology - René Gauducheau, Bd J. Monod, Nantes, Saint Herblain Cedex 44805 France; Biopatholgy department, Integrated Centre for Oncology - René Gauducheau, Bd J. Monod, Nantes, Saint Herblain Cedex 44805 France

**Keywords:** Age, Breast cancer, Gene-expression signatures, Genomics

## Abstract

**Background:**

Breast cancer biological characteristics change as age advances. Today, there is a lack of knowledge regarding age-specific molecular alterations that characterize breast tumours, notably in elderly patients. The vast majority of studies that aimed at exploring breast cancer in function of age are based on clinico-pathological data. Gene-expression signatures (GES), which in some ways capture biological information in a non-reductionist manner, represent powerful tools able to explore tumour heterogeneity.

**Methods:**

Twenty-five GES were used for functional annotation of breast tumours in function of age: five for molecular subtyping, seven for immune response, three for metabolism, seven for critical pathways in cancer and three for prognosis. Affymetrix® genomics datasets were exclusively used to avoid cross-platform normalization issues. Available corresponding clinico-pathological data were also retrieved and analysed.

**Results:**

Fifteen publicly available datasets were pooled for a total of 2378 breast cancer patients (whole cohort), out of whom 1413 were of Caucasian origin. Three age groups were defined: ≤ 40 years (AG1), > 40 to < 70 years (AG2) and ≥ 70 years (AG3). We confirmed that age influenced the incidence of molecular subtypes. We found a significant growing incidence of luminal B and a decreasing kinetics for basal-like in function of age. We showed that AG3 luminal B tumours were less aggressive than AG1 luminal B tumours based on different GES (iron metabolism, mitochondrial oxidative phosphorylation and reactive stroma), recurrence score prognostic GES and histological grade (SBR). Contrary to tumours of young patients, tumours of elderly patients concentrated favourable GES scores: high oestrogen receptor and mitochondrial oxidative phosphorylation, low proliferation, basal-like, glycolysis, chromosomal instability and iron metabolism, and low GES prognostic scores (van’t Veer 70-GES, genomic grade index and recurrence score).

**Conclusions:**

Functional annotation of breast tumours by means of 25 GES demonstrated a decreasing aggressiveness of breast tumours in function of age. This strategy, which can be strengthened by increasing the number of representative GES to gain more insight into biological systems involved in this disease, provides a framework to develop rational therapeutic strategies in function of age.

**Electronic supplementary material:**

The online version of this article (doi:10.1186/s12920-015-0153-6) contains supplementary material, which is available to authorized users.

## Background

Breast cancer heterogeneity makes it difficult to bring personalized medicine into the clinic. Since many years, research aimed at deciphering molecular presentation of this disease to identify subgroups of patients with clinical significance, such as prognosis or response to therapy, able to optimize patient management. The era of large-scale science, which is linked both to recent technological advances and to the availability of full genetic information, has boosted the research for new biomarkers and molecular subtyping. In 2000 and 2001, Perou and Sorlie defined five breast cancer molecular subtypes based on gene-expression profile homologies of an intrinsic gene list that included 427 unique genes: basal-like, HER2-E, luminal A, luminal B, and normal breast-like [1, 2]. They showed that these subgroups of tumours were linked to histology, and their corresponding markers, different natural histories, response to treatment, and prognosis. Since these seminal studies, in breast cancer research, numerous gene-expression signatures (GES) with different purposes (molecular subtyping, biological pathway exploration, prognosis) emerged. Briefly, GES are composed of combinations of genes ranging from few to hundreds, which in some ways capture biological information in a non-reductionist and more informative manner. For example, researchers demonstrated that p53 GES predicted outcome better than p53 mutation status alone, and others showed that a signature of MYC activation, which better reflected MYC transcriptional output, was more informative than MYC gene expression alone [3, 4]. In breast cancer, many studies demonstrated that GES and immunohistochemistry (IHC) were not concordant, notably for triple-negative tumours [5]. Integrated studies, combining clinico-pathological, IHC and transcriptomic data, demonstrated that GES were powerful tools to molecularly dissect breast tumours [6].

It is well recognized that breast cancer biological characteristics change as age advances. Today, there is a lack of knowledge regarding gene-expression molecular dissection of breast cancer tumours in function of age, notably in elderly patients [7]. Age-specific molecular alterations that characterize breast tumours remain to be elucidated. The vast majority of studies that aimed at exploring breast cancer in function of age are based on clinico-pathological data. Three recent works determined intrinsic molecular subtypes by means of PAM50 GES in function of age, but limited their analyses only to this subtyping GES [8–10]. In this study, we conducted functional annotation of breast cancer tumours divided in three age groups (≤40, > 40 to < 70 and ≥ 70 years) by means of 25 GES. Available clinico-pathological data (oestrogen receptor [ER] status, HER2 status, nodal status [N], Scarff-Bloom-Richardson [SBR] histological grade, tumour size and evolution data) were analysed in parallel. Because of a possible ethnic bias, analyses were done twice, on a whole cohort composed of patients of mixed geographic origins, and on a Caucasian subcohort; numbers of patients were 2378 and 1413, respectively.

Functional annotation by means of 25 GES tested in this study demonstrated a decreasing aggressiveness of breast tumours in function of age based on continuous GES scoring. Furthermore, we showed that luminal B tumour of elderly patients were less aggressive than luminal B tumour of young patients.

## Methods

### Data selection

We exclusively looked for publicly available breast cancer Affymetrix® genomic datasets associated with clinico-pathological information, including age at diagnosis, prognosis and geographic origin of the cohorts in repositories such as Gene Expression Omnibus (GEO), ArrayExpress, author's individual web pages, and in articles, selecting those with a medium to large sample size [11, 12]. Among other things, our study aimed at exploring molecular subtype distribution, which is known to vary in function of population origin [10, 13–16]. For this reason, we selected a Caucasian cohort from the whole population. However, precise origin of the patients was exceptionally indicated in clinico-pathological characteristics associated with genomic data. So, we selected European cohorts and three non-European ones (E_TABM_158, GSE7849 and GSE17907), for whom ethnicity was reported; non-Caucasian patients were excluded from these three studies. According to the cohorts’ country of origin, we supposed that they were composed of a large majority of Caucasian women. Patients who received neoadjuvant chemotherapy and microdissected samples were not included.

### Data pre-processing

Data pre-processing and normalization were described elsewhere [17] and are summarized in Additional file 1.

### Gene-expression signatures

Twenty-five GES were selected for functional annotation of breast cancer tumours. Five GES were used for breast cancer molecular subtyping: PAM50, ER, molecular apocrine, basal-like and claudin-CD24. Seventeen were linked to biological processes of importance or cell types: immune response (B-cell, interferon [IFN], interleukin-8 [IL-8], MHC-1, MHC-2, T-cell, M2-macrophages/M1-macrophages enrichment [M2/M1]), metabolism (adipocytes, glycolysis, iron [IRGS]) and critical biological pathways in cancer (chromosomal instability [CIN], ERBB2, HOXA, mitochondrial oxidative phosphorylation [MITO/OXPHOS], proliferation, reactive stroma, VEGF). Finally, three prognostic GES were also used: van’t Veer 70-GES, recurrence score (RS) and genomic grade index (GGI). Complete GES list, methods and references are briefly described in Additional file 1.

### Statistical analysis

Evolution analysis based on pejorative events (local relapse, metastatic relapse or death), metastatic relapse alone (MFS) and overall survival (OS) were estimated by the Kaplan-Meier method and compared between the age groups by the Log-rank test.

Mantel-Haenszel chi-square trend test was used to analyse relations between clinico-pathological characteristics (ER, HER2, SBR histological grade, nodal status, tumour histological size) and ordered age categories. In addition, unordered multinomial logistic regression (UMLR) was used for PAM50 subtype distribution and SBR histological grade in luminal B tumours in function of continuous age. Kinetics was determined by the value of odds ratio (OR). One way analysis of variance (ANOVA), followed by Tukey post-hoc test for pairwise comparisons in case of significance, was used to compare continuous variables between the three age groups. GES subtyping and scoring were done on patients for whom at least 75 % of GES genes were available in their expression data (Additional file 1). Twenty four continuous GES (all GES except PAM50) score correlations were illustrated with a correlation plot along with the dendrogram corresponding to average-linkage hierarchical clustering algorithm of GES with Pearson correlation distance measure.

We considered a two-sided *p*-value of less than 0.05 to be statistically significant (for multiple comparisons, Bonferroni correction was applied); the same level was used for significance analysis of microarrays (SAM) q-value. Mantel-Haenszel chi-square trend test, UMLR, hierarchical clustering algorithm and SAM method were done with R software (version 3.0.2) and packages; coin, VGAM, amap and samr, respectively. STATA® was used for survival analyses (version 12.0).

## Results

### Included patients

We exclusively focused on Affymetrix® genomic datasets to avoid cross-platform normalization issues. Fifteen publicly available datasets were pooled for a total of 2378 patients, out of whom 1413 were of Caucasian origin (Table 1). Three patients’ age groups were defined: ≤ 40 years (AG1), > 40 to < 70 years (AG2) and ≥ 70 years (AG3). Numbers of patients in each age group for both populations are displayed in Tables 2 and 3.

**Table 1:** Cohorts included in our study

n	Study code	Affymetrix® array	References	Patients n	Geographic origins	Caucasian n
1	E_TABM_158	HG-U133A	[18]	112	USA	81
2	GSE2603	HG-U133A	[19]	82	USA	0
3	GSE4922	HG-U133A + B	[20]	249	Sweden	249
4	GSE6532	HG-U133A + B + Plus2	[21]	401	UK, Sweden	401
5	GSE7378	HG-U133A	[22]	54	USA	0
6	GSE7390	HG-U133A	[23]	198	Sweden, France, UK	198
7	GSE7849	HG-U95A	[24]	75	USA	58
8	GSE9195	HG-U133Plus2	[25]	77	UK, Sweden	77
9	GSE16391	HG-U133Plus2	[26]	55	International	0
10	GSE17907	HG-U133Plus2	[27]	49	France, Tunisia	43
11	GSE19615	HG-U133Plus2	[28]	115	USA	0
12	GSE20685	HG-U133Plus2	[29]	296	Taiwan	0
13	GSE21653	HG-U133Plus2	[30]	265	France	265
14	GSE25055	HG-U133A	[31]	309	USA	0
15	GSE45255	HG-U133A	[32]	41	Belgium	41
			Total	2378		1413

*NA* not available

**Table 2:** Clinico-pathological characteristics according to the three patients’ age groups of the whole cohort

Patients’ group (years and [n])		n 2378	≤40 (*n* = 345)	40 < x < 70 (*n* = 1667)	≥70 (*n* = 366)	*p*-value
Age (years)						
	Median (IQR)	54 (45–64)	37 (34–39)	54 (47–61)	74 (72–80)	
ER status						
	Positive	1671 (71 %)	199 (58 %)	1170 (71 %)	302 (83 %)	<0.0001
	Negative	681 (29 %)	143 (42 %)	477 (29 %)	61 (17 %)	
HER2 status						
	Positive	136 (14 %)	28 (19 %)	89 (13 %)	19 (17 %)	0.4862
	Negative	808 (86 %)	121 (81 %)	592 (87 %)	95 (83 %)	
Nodal status						
	Positive	882 (43 %)	130 (49 %)	603 (42 %)	149 (44 %)	0.4010
	Negative	1172 (57 %)	138 (51 %)	847 (58 %)	187 (56 %)	
SBR grade						
	1	302 (17 %)	15 (6 %)	223 (17 %)	64 (22 %)	<0.0001
	2	770 (42 %)	83 (36 %)	561 (44 %)	126 (43 %)	
	3	742 (41 %)	134 (58 %)	504 (39 %)	104 (35 %)	
Tumour size						
	≤20 mm	626 (46 %)	57 (44 %)	458 (48 %)	111 (42 %)	0.3993
	>20 mm	723 (54 %)	72 (56 %)	499 (52 %)	152 (58 %)	

Abbreviations: *ER* oestrogen receptor, *IQR* interquartile range, *SBR* Scarff-Bloom-Richardson histological grade

**Table 3:** Clinico-pathological characteristics according to the three patients’ age groups of the Caucasian cohort

Patients’ group (years and [n])		n 1413	≤40 (*n* = 166)	40 < x < 70 (*n* = 974)	≥70 (*n* = 273)	*p*-value
Age (years)						
	Median (IQR)	57 (47–67)	36 (33–38)	56 (49–62)	74 (72–80)	
ER status						
	Positive	1054 (76 %)	90 (55 %)	735 (76 %)	229 (85 %)	<0.0001
	Negative	341 (24 %)	73 (45 %)	227 (24 %)	41 (15 %)	
HER2 status						
	Positive	85 (23 %)	25 (36 %)	46 (19 %)	14 (22 %)	0.0407
	Negative	292 (77 %)	44 (64 %)	198 (81 %)	50 (78 %)	
Nodal status						
	Positive	498 (36 %)	62 (38 %)	319 (33 %)	117 (46 %)	0.0263
	Negative	889 (64 %)	100 (62 %)	651 (67 %)	138 (54 %)	
SBR grade						
	1	254 (19 %)	12 (8 %)	182 (20 %)	60 (24 %)	<0.0001
	2	571 (44 %)	57 (36 %)	403 (45 %)	111 (44 %)	
	3	482 (47 %)	88 (56 %)	311 (35 %)	83 (33 %)	
Tumour size						
	≤20 mm	537 (49 %)	46 (47 %)	388 (50 %)	103 (45 %)	0.4794
	>20 mm	567 (51 %)	52 (53 %)	390 (50 %)	125 (55 %)	

Abbreviations: *ER* oestrogen receptor, *IQR* interquartile range, *SBR* Scarff-Bloom-Richardson histological grade

### Clinico-pathological characteristics according to patients’ age

Associations with known clinico-pathological characteristics are shown in Tables 2 and 3. A significant difference was found for ER in function of age group (*p* < 0.0001) associated with a positive kinetics. This result was in line with current knowledge [33, 34]. A significant difference was found for HER2 in the Caucasian cohort (*p* = 0.0407), with a decreasing kinetics for HER2+ in function of age group. In Caucasian cohort, an increasing kinetics was found for positive nodal status (N+) according to age group (*p* = 0.0263) (Table 3). SBR histological grade distribution was different in function of age in the two cohorts (*p* < 0.0001): SBR3 decreased compared with SBR1 from young to old patients in both cohorts. Histological size, dichotomized based on 20 mm cut-off, did not vary. However, a difference was shown when continuous size of tumour was used in whole cohort (*p* = 0.0337) and even in Caucasian one (*p* = 0.0354). Histological size was higher in AG3 compared to AG2 for Caucasian one (*p* = 0.0336). Histological size and N+ status positive kinetics in function of age were also concordant with current knowledge [33, 35, 36].

### Evolution analysis

**Fig. 1:**
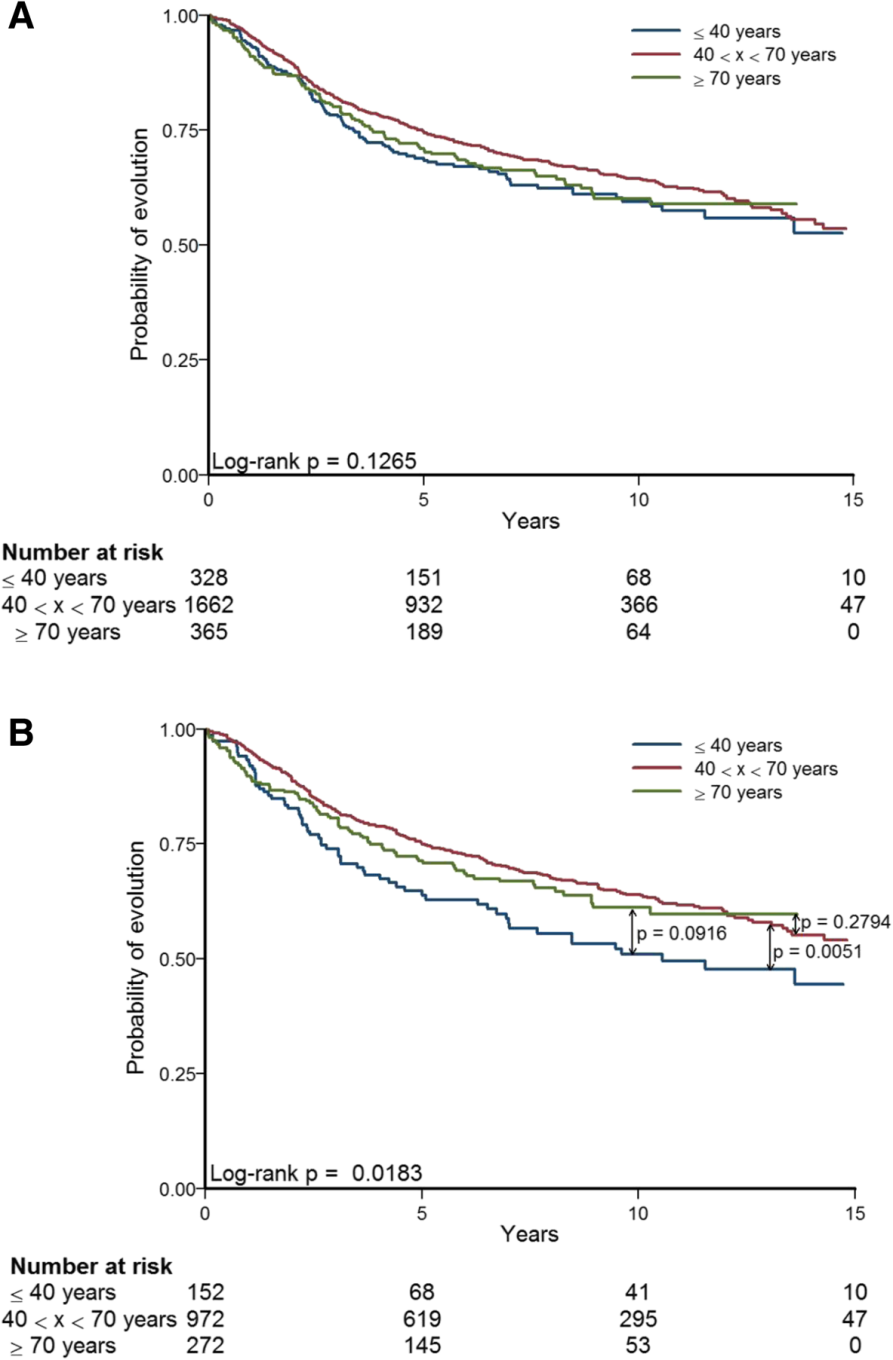
Kaplan-Meier curves in function of the three patients’ age groups. **a** Whole cohort. **b** Caucasian cohort

In the whole cohort, no difference in evolution rates was observed between the three patients’ age groups (*p* = 0.1265) (Fig. 1a). Similar results were found with MFS and OS as outcomes (Additional file 2). On the contrary, in Caucasian cohort, young patients displayed a worse prognosis compared to patients of the intermediate group (*p* = 0.0051); no difference was found between elderly patients and patients of the intermediate group (*p* = 0.2794) (Fig. 1b). We have to underline that evolution analyses in elderly cancer patients must be interpreted cautiously because undertreatment and comorbidities in older patients distort them. This point will be discussed later.

### Molecular dissection by means of 25 GES

**Fig. 2:**
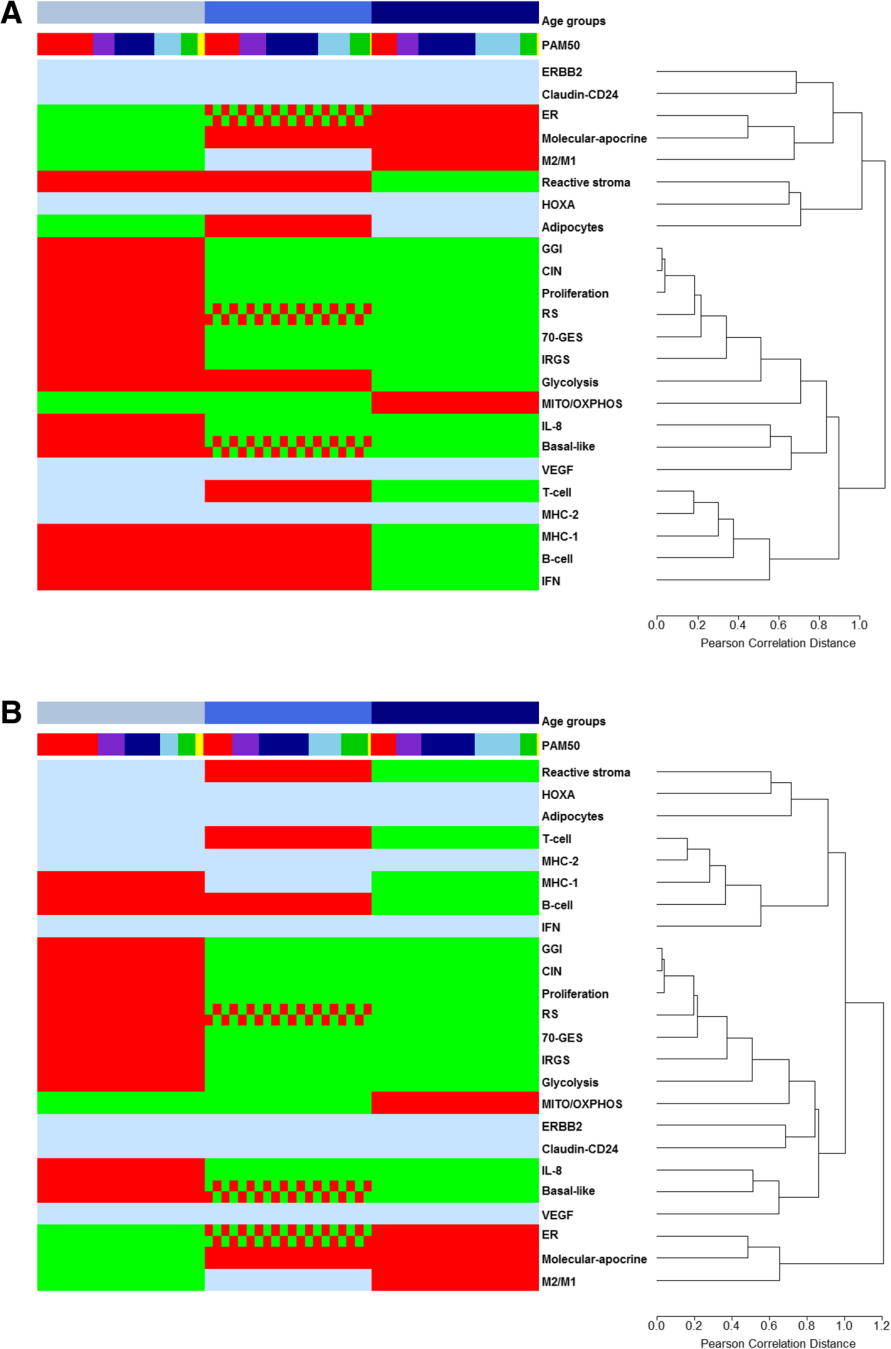
Molecular dissection of breast cancer tumours in function of age. Average hierarchical clustering and heatmap showing the segregation of three age groups as a function of 24 GES scores. **a** Whole cohort. **b** Caucasian cohort. These figures are illustrations of table 4 statistical analyses. First row presents the three age groups: ≤ 40 years (**a**, *n* = 345; **b**, *n* = 166) (sky blue), 40 < x < 70 years (**a**, *n* = 1667; **b**, *n* = 974) (medium blue) and ≥ 70 years (**a**, *n* = 366; **b**, *n* = 273) (dark blue), from left to right. Other rows, from top to bottom, present PAM50 GES subtyping (basal-like (red); HER2-E (purple); luminal A (dark blue); luminal B (sky blue); normal breast-like (green); unclassified (yellow)) and GES scores in function of age (green: low score; red and green grid pattern: intermediate score; red: high score; sky blue: not significant or not interpretable)

Nineteen GES out of 24 continuous GES showed significant differences (ANOVA and Tukey post-hoc test) in function of age. ERBB2, HOXA, VEGF, Claudin-CD24 and MHC-2 GES were never significant whatever the cohort. GES molecular dissection results for the whole cohort and Caucasian one are displayed in Table 4, Fig. 2 and Additional file 3.

**Table 4:** Continuous GES analyses interpretation in function of three age groups

GES name	Whole cohort						Caucasian cohort					
	*p*-value		*p*-value		Results		*p*-value		*p*-value		Results	
		1 vs 2	1 vs 3	2 vs 3				1 vs 2	1 vs 3	2 vs 3		
Molecular subtyping												
ER	<0.0001	<0.0001	<0.0001	0.0014	1 < 2 < 3		<0.0001	<0.0001	<0.0001	0.0096	1 < 2 < 3	
Molecular-apocrine	<0.0001	<0.0001	<0.0001	0.3376	1 < 2 ≈ 3		<0.0001	<0.0001	<0.0001	0.5479	1 < 2 ≈ 3	
Basal-like	<0.0001	<0.0001	<0.0001	<0.0001	1 > 2 > 3		<0.0001	<0.0001	<0.0001	0.0006	1 > 2 > 3	
Claudin-CD24	0.8457				NS		0.9813				NS	
Immune response												
B-cell	<0.0001	0.7488	<0.0001	<0.0001	1 ≈ 2 > 3		0.0001	0.9398	0.0053	0.0001	1 ≈ 2 > 3	
T-cell	0.0016	0.7797	0.0667	0.0010	2 > 3	1 ≈ 2 and 1 ≈ 3	0.0204	0.9736	0.1891	0.0153	2 > 3	1 ≈ 2 and 1 ≈ 3
MHC-1	0.0175	0.8578	0.0368	0.0215	1 ≈ 2 > 3		0.0300	0.6309	0.0471	0.0589	1 > 3	1 ≈ 2 and 2 ≈ 3
MHC-2	0.0133	0.0616	0.9987	0.0623	NS		0.0486	0.1769	0.9901	0.1133	NS	
M2/M1	0.0136	0.3213	0.0113	0.0616	1 < 3	1 ≈ 2 and 2 ≈ 3	0.0429	0.2421	0.0341	0.2445	1 < 3	1 ≈ 2 and 2 ≈ 3
IFN	0.0067	0.3113	0.0059	0.0326	1 ≈ 2 > 3		0.0633				NS	
IL-8	0.0004	0.0091	0.0003	0.0917	1 > 2 ≈ 3		<0.0001	<0.0001	<0.0001	0.3518	1 > 2 ≈ 3	
Metabolism												
Adipocytes	0.0118	0.0140	0.5359	0.2931	1 < 2	1 ≈ 3 and 2 ≈ 3	0.2272				NS	
Glycolysis	0.0044	0.1734	0.0032	0.0418	1 ≈ 2 > 3		0.0037	0.0112	0.0031	0.4653	1 > 2 ≈ 3	
IRGS	0.0013	0.0012	0.0047	0.9587	1 > 2 ≈ 3		0.0122	0.0458	0.0085	0.3879	1 > 2 ≈ 3	
Critical biological pathways in cancer												
CIN	<0.0001	<0.0001	0.0003	0.9997	1 > 2 ≈ 3		<0.0001	<0.0001	0.0012	0.7143	1 > 2 ≈ 3	
ERBB2	0.0771				NS		0.0473	0.9297	0.1280	0.0506	NS	
HOXA	0.7895				NS		0.5566				NS	
MITO/OXPHOS	<0.0001	0.2278	<0.0001	0.0006	1 ≈ 2 < 3		0.0013	0.8029	0.0097	0.0018	1 ≈ 2 < 3	
Proliferation	<0.0001	<0.0001	0.0003	0.9989	1 > 2 ≈ 3		<0.0001	<0.0001	0.0013	0.9542	1 > 2 ≈ 3	
Reactive stroma	<0.0001	0.1912	0.0487	<0.0001	1 ≈ 2 > 3		0.0001	0.1684	0.3841	0.0001	2 > 3	1 ≈ 2 and 1 ≈ 3
VEGF	0.4340				NS		0.0743				NS	
Prognosis												
70-GES	<0.0001	<0.0001	0.0001	0.9441	1 > 2 ≈ 3		<0.0001	<0.0001	<0.0001	0.9839	1 > 2 ≈ 3	
GGI	<0.0001	<0.0001	0.0008	0.9303	1 > 2 ≈ 3		0.0004	0.0003	0.0031	0.9902	1 > 2 ≈ 3	
RS	<0.0001	<0.0001	<0.0001	0.0006	1 > 2 > 3		<0.0001	<0.0001	<0.0001	0.0183	1 > 2 > 3	

1: ≤ 40 years; 2: 40 < x < 70 years; 3: ≥ 70 years; *ER* oestrogen receptor, *IFN* interferon, *IRGS* iron regulatory gene signature, *CIN* chromosomal instability, *MITO/OXPHOS* mitochondrial oxidative phosphorylation, *NS* not significant (*p* > 0.05), *70-GES* van’t Veer and colleagues prognostic GES, *GGI* genomic grade index, *RS* recurrence score

**Fig. 3:**
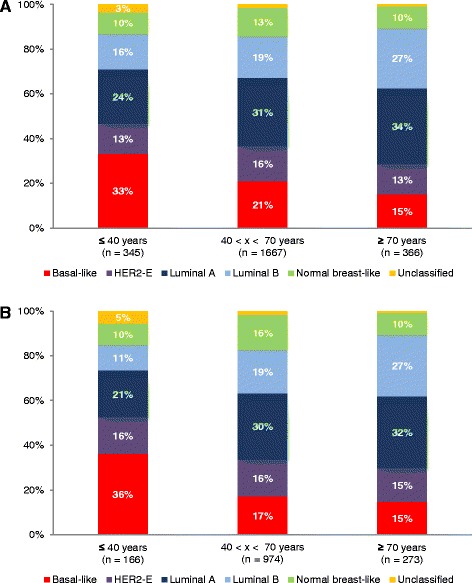
Distribution of the five PAM50 molecular subtypes in function of age. **a** Whole cohort. **b** Caucasian cohort

**Fig. 4:**
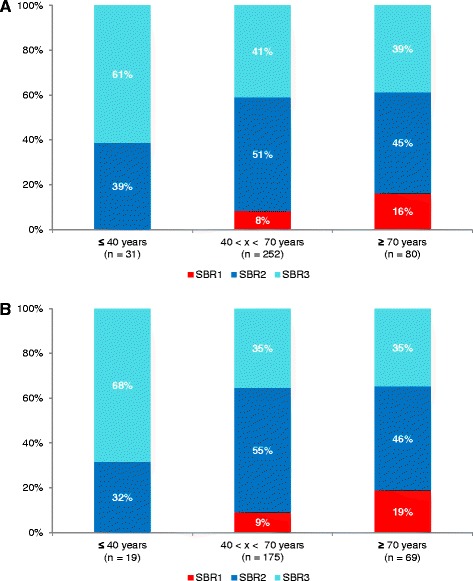
SBR histological grade distribution in luminal B tumours in function of age. **a** Whole cohort. **b** Caucasian cohort

A total of 2378 patients were subtyped by means of PAM50 GES. Subtype distributions are displayed in Fig. 3. Statistical analyses showed a significant growing incidence of luminal B in function of age (trend test: *p* = 0.0004 and *p* < 0.0001 for whole cohort and Caucasian cohort, respectively). In order to strengthen this result based on ordered age categories, we performed UMLR, which uses continuous age. UMLR result was concordant with trend test (OR > 1; *p* < 0.05 for both cohorts). Only trend test was significant for growing incidence of luminal A for whole cohort (*p* = 0.0092). A decreasing kinetics for basal-like was found for the whole cohort (trend test: *p* < 0.0001; UMLR: OR < 1; *p* < 0.0060) and for the Caucasian one (trend test: *p* < 0.0001; UMLR: OR < 1; *p* < 0.0005). No other age-related kinetics was found for other subtypes. It is a bit surprising to notice an increasing incidence of luminal B, which is known to be a bad prognostic subtype, because breast cancer in advanced age has been associated with a slightly increased probability of favourable tumour biology [33]. Aggressive subtypes should be underrepresented and, on the contrary, less aggressive subtypes should be overrepresented; as we found, basal-like kinetics was in agreement with this. One hypothesis could be that luminal B tumours of elderly patients are less aggressive than those of younger patients. To explore this hypothesis, we evaluated SBR histological grade distribution in luminal B tumours in function of age (Fig. 4). We chose to conduct analysis with only SBR1 (less aggressive) and SBR3 (high aggressive) because clinical interpretation of SBR2 is complicated. Analyses showed that SBR1 percentage increased and SBR3 decreased in function of age in luminal B tumours in whole cohort (trend test: *p* = 0.0168; UMLR: OR = 0.96, *p* = 0.0035) and even in Caucasian cohort (trend test: *p* = 0.0259; UMLR: OR = 0.95, *p* = 0.0042), which was consistent with our hypothesis. Furthermore, GES analyses of patients belonging to AG1 and AG3 showed an “AG1 > AG3” profile for reactive stroma, IRGS and RS, and an “AG1 < AG3” profile for MITO/OXPHOS, which testifies to the aggressiveness of AG1 luminal B tumours compared to AG3 luminal B tumours, in both cohorts or in the whole cohort (Additional file 4). Evolution analysis of luminal B patients in function of the three age groups did not show any difference (whole cohort: *p* = 0.7717; Caucasian cohort: *p* = 0.3969). For reasons cited above (evolution analyses biases in elderly) and discussed later, this result did not invalidate our hypothesis.

ER GES showed an increasing kinetics in function of age groups as demonstrated above by means of IHC-measured ER. Molecular apocrine scores were the smallest in young patients and higher in the two other age groups suggesting an increased androgen receptor signalling in these last ones. This finding was concordant with some studies based on IHC, which indicated a higher rate of apocrine carcinoma in elderly and a correlation with menopausal status [37, 38]. Molecular apocrine GES scores often clustered with ER (Additional file 3). Basal-like GES scores were in concordance with basal-like PAM50 subtyping; the same decreasing kinetics was found in function of age groups.

Molecular dissection of the three age groups by means of seventeen GES linked to biological processes of importance or cell types showed concordant results in both cohorts for B-cell (AG1 ≈ AG2 > AG3), T-cell (AG2 > AG3), IL-8, IRGS, proliferation and CIN (AG1 > AG2 ≈ AG3), MITO/OXPHOS (AG1 ≈ AG2 < AG3), and M2/M1 (AG1 < AG3) (Table 4). When focusing only on the two extreme age groups, there were more concordant results between the two cohorts: basal-like, B-cell, MHC-1, IL-8, CIN, proliferation, glycolysis and IRGS displayed an “AG1 > AG3” profile, whereas ER, molecular apocrine, MITO/OXPHOS and M2/M1 displayed an “AG1 < AG3” profile.

Immune response (IR) evaluated by B-cell, MHC-1 and IL-8 GES was lower in elderly patients for both cohorts. This result could be due to immunosenescence, which was demonstrated in different transcriptomic works, rather than local low IR or local immunosuppression [39, 40]. Here, we showed that IL-8 and basal-like always clustered together (Additional file 3). IL-8, member of the CXC chemokine family of angiogenesis/inflammation-related chemokines, is overexpressed in breast cancer, is linked to bad prognosis, has a direct role in angiogenesis and is involved in cancer stem-like cells (CSC) regulation [41, 42]. Because CSC are associated with basal-like subtype in breast cancer, IL-8/basal-like cluster might indirectly illustrate IL-8/CSC biological pathway [43]. M2/M1 showed an opposite result: high score in elderly patients (AG1 < AG3). In a recent study, which aimed at subtyping triple negative breast cancer (TNBC) tumours, we found that IR was associated with good prognosis in a basal-like- and claudin-low-enriched TNBC subtype [6]. On the contrary, M2/M1 high scores, meaning enrichment in M2 pro-tumourigenic macrophages were associated with a bad prognosis for pure basal-like TNBC subtype. M2/M1 GES applied on TNBC and PAM50 basal-like patients of our study failed to reach significance in whole cohort (*p* = 0.3722; AG1 = 36, AG2 = 149, AG3 = 21) and in Caucasian cohort (*p* = 0.3166; AG1 = 8, AG2 = 46, AG3 = 12). This GES needs further investigations to define its scope of use in breast cancer.

Elderly patients had the lowest stromal reaction for the whole cohort and a lower stromal reaction compared to the intermediate age group for Caucasian one. This metagene captured different clinico-biological information. Its pattern of expression is similar to that of mammospheres and epithelial to mesenchymal signature metagene and is associated with chemoresistance [44]. A weak correlation was found for reactive stroma and adipocytes GES scores (Additional file 3).

70-GES and GGI scoring displayed an “AG1 > AG2 ≈ AG3” profile and RS scoring an “AG1 > AG2 > AG3” profile, respectively. In the first case, AG1 was the worst prognostic group. In the second case, a decreasing prognostic kinetics was shown, starting with AG1 as the worst prognosis age group and ending with AG3 as the best prognosis age group. RS score results might confirm the negative effect of different and less effective therapeutic management compared with younger patients on survival of elderly patients, which, despite best prognostic scores, is not significantly different from AG1 patients’ survival, neither in whole cohort, nor in Caucasian one (while, in the latter cohort, AG2 patients’ survival is significantly better than AG1 patients’) (Fig. 1). Furthermore, 70-GES, GGI and RS clustered strongly with proliferation (Additional file 3). This can be explained by the fact that proliferation is the common driving force of these prognostic GES [45, 46].

GES analyses interpretation in function of ER status in the three age groups confirmed that proliferation (proliferation GES, 70-GES, GGI and RS) was of no value in predicting aggressiveness and prognosis in ER-negative breast cancer tumours (Additional file 5) [45].

SAM analysis identified 1116 genes with statistically significant changes in expression between the two extreme age-groups, AG1 and AG3: 432 overexpressed and 684 underexpressed genes in AG3 [47]. GO biological process enrichment analyses of these two gene lists by means of ToppGene web tool showed that cell cycle and cell migration, which are considered as basal-like hallmark, characterized AG1, and that oxidation-reduction process and lipid metabolism, which are considered as luminal hallmark, characterized AG3 (Additional file 6) [48, 49]. Although these non-hypothesis-driven results might be seen as too overly broad, they corroborated GES molecular dissection of breast tumours in function of age.

## Discussion

Functional annotation may be separated into two ways of analyses: a quantitative one by means of GES, which assigns scores to patients, and a qualitative one based on gene lists, which assigns a “Gene Ontology” enrichment score to a cluster representative gene list [49].

In this work, we focused on GES scoring and used “Gene Ontology” enrichment score to corroborate our findings. Functional annotation according to the three age groups by means of clinico-pathological data was done to confirm that our cohort was representative of breast cancer population. Because of a possible ethnic bias, analyses were done twice: on the whole cohort and on the Caucasian one. Finally, there were only slight differences between whole cohort results and Caucasian results. Statistical comparisons could not be done because these cohorts were not independent.

GES used in our study can be classified into different categories: molecular subtyping (*n* = 5), immune response (*n* = 7), metabolism (*n* = 3), critical biological pathways in cancer (*n* = 7) and prognosis (*n* = 3). One question that emerged is potential biological redundancy between GES. Except for prognosis GES (70-GES, RS and GGI), CIN and PAM50, which are enriched in proliferation genes, other GES captured different biological information based on varying pathways. Numbers of genes in common between these last GES are equal or close to zero (Additional file 7). Except M2/M1 GES all other IR GES have no genes in common. However it is well known that different combination of genes can capture the same biological information. In order to look for overlapping biological information, we compared GO enrichment terms (biological process tree) linked to each GES’s gene list (Fisher's exact test; *p* < 0.01). Results are displayed in Additional files 8 and 9. Only a few remarks can be made. In some cases, limited GES similarities exist and are concordant with biological knowledge. Biological process similarities emerged for IR GES (B-cell, IFN, IL-8, MHC-1, MHC-2 and T-cell) and as expected for GES enriched in proliferation genes (Proliferation, GGI, CIN, PAM50, RS and 70-GES). Another biological process cluster is found for ER, RS, Molecular apocrine and PAM50. RS and PAM50 are strongly dependent on ER pathway. Out of 16 cancer genes included in RS GES, four belong to ESR1 cluster: *ESR1*, *PGR*, *BCL2* and *SCUBE2*. Link between ER and molecular apocrine GES is explained by the fact that molecular apocrine subtype has a gene expression profile resembling that of ER-positive tumours [50, 51]. Our strategy might be improved by enriching GES list. Focusing on other and non-redundant critical biological pathways in cancer and increasing breast tumour transcriptome deconvolution will certainly enhance molecular dissection performance.

Molecular dissection by means of GES tested in this study confirmed that breast cancer tumours of young patients were more aggressive than tumours of older patients. Furthermore, we showed that tumour aggressiveness decreased regularly in function of age groups based on continuous GES scoring. Finally, tumours of elderly patients concentrated favourable GES scores: high ER, MITO/OXPHOS, low proliferation, basal-like, glycolysis, CIN and IRGS, and low GES prognostic score (70-GES, GGI and RS). These biological features were not linked to favourable disease evolution, but this result must be interpreted cautiously in elderly patients. Undertreatment in older patients with breast cancer is known to have a strong negative effect on survival [36, 52–54]. Furthermore, in our study, evolution criteria were: local relapse, metastatic relapse, or death. Due to non-cancer-related death linked to comorbidity, which we could not identify, this last pejorative event is likely higher in elderly patients and may significantly skew evolution analyses [55]. One study reported that 44 % of undertreated elderly patients died during follow-up period without disease recurrence [54].

We confirmed that age influences the incidence of molecular subtypes and found a significant growing incidence of luminal B and a decreasing kinetics for basal-like in function of age. As we underlined, luminal B kinetics was surprising, but concordant with Jenkin’s and de Kruijf’s studies [8, 9]. On the contrary, Sweeney and colleagues found an opposite result [10]. In this work, we showed that AG3 luminal B tumours were less aggressive than AG1 luminal B tumours based on SBR histological grade and four GES (IRGS, MITO/OXPHOS, reactive stroma and RS). Our results are concordant with an IHC study conducted by Morrison and colleagues [56]. They found high proliferation (Ki67 > 14 %), high mutated P53 (≥10 %) and high Nottingham grade in young luminal B (≤40 years) compared to older luminal B patients (≥50 years). At this time, it is premature to definitively conclude and additional studies will be necessary to confirm these results.

In our study, the GES molecular dissection with the most complicated results interpretation were those provided by IR GES. An “AG1 > AG3” profile was found for B-cell, MHC-1 and IL-8, an “AG2 > AG3” for T-cell in both cohorts, and an “AG1 ≈ AG2 > AG3” for IFN for whole cohort. Aside from M2/M1 scores, when focusing only on the two extreme age groups, these results demonstrated a high IR in young patients and a low IR in elderly patients. Immunity is known to play a dual role in the complex interactions between tumours and the host. In established cancer, some immune cells are known to induce anti-tumoural effects (immunosurveillance) (NK cells, CD8+ T cells, Th1 cells, dendritic cells 1, M1 macrophages…), and others, pro-tumoural effects (myeloid-derived suppressor cells, CD4+ T cells, Th2 cells, dendritic cells 2, M2 macrophages…) [57-59]. A dynamic model, called “immunoediting”, more appropriately emphasizes the dual roles of immunity and describes tumour and immune system interactions in a chronological three phase process: elimination (immunosurveillance), dynamic equilibrium and escape [60, 61]. The disruption of this equilibrium results from Darwinian selection of a new population of tumour clones able to escape from immune detection and/or elimination, allowing tumour progressive growth and dissemination. A second level of complexity is due to an age-dependent general decline of immune function that has been termed immunosenescence, which is associated with high circulating level of pro-inflammatory cytokines. As a consequence of these facts, most obvious, reductionist and parsimonious questions which can be asked are: In elderly patients, is low intra-tumoural IR principally due to a local effect of immunosenescence? In young patients with high intra-tumoural IR, does bad prognosis depend in part upon IR escape as proposed in immunoediting dynamic model? In answering these questions, we will be able to progress in the selection of immunotherapy strategies in function of age.

## Conclusions

Our study demonstrated that a panel of GES may be used to decipher the heterogeneity of breast cancer in function of age and represented a preliminary work. A GES list including most of relevant biological pathways in breast cancer will certainly help to gain more insight into biological systems involved in this disease and will provide a framework to develop rational therapeutic strategies based on meaningful subtyping. We believe that GES are efficient tools able to dissect tumours because they do not depend on only one prototypic marker, but are composed of a combination of genes involved and/or correlated to a particular molecular pathway, and for this reason, capture more efficiently biological information.

### Availability of supporting data

The datasets, listed in Table 1, used in this article are publicly available on EBI website (www.ebi.ac.uk) for E_TABM_158, and on GEO website (www.ncbi.nlm.nih.gov/geo) for all other cohorts.

## Additional files

Additional file 1:
**GES listing, methods and references.** (PDF 114 kb) Additional file 2:
**Metastasis-free survival (MFS) and overall survival (OS) analyses.** MFS: 2A1: whole cohort; 2A2: Caucasian cohort. OS: 2B1: whole cohort; 2B2: Caucasian cohort. (PDF 230 kb) Additional file 3:
**GES scoring of breast cancer in function of age.** Correlation matrices show Pearson correlation coefficients between continuous GES (red indicates a positive correlation; blue, a negative correlation) and dendrograms show mutual relationships of all signatures (scores were used for average-link hierarchical clustering using the Pearson correlation as a distance metric). 2A1: AG1, whole cohort; 2A2: AG2, whole cohort; 2A3: AG3 whole cohort; 2B1: AG1, Caucasian cohort; 2B2: AG2, Caucasian cohort; 2B3: AG3, Caucasian cohort. (PDF 1689 kb) Additional file 4:
**GES analyses differentiating AG1 and AG3 luminal B patients.** AG1 > AG3 means that AG1 GES score is superior to AG3 GES score, and inversely. (PDF 57 kb) Additional file 5:
**Continuous GES analyses interpretation in function of ER status in the three age groups.** (PDF 75 kb) Additional file 6:
**GO biological process enrichment analyses of most differentially expressed genes between AG1 and AG3.** Head of columns represents SAM gene lists used as ToppGene inputs. The ten first significant biological processes are displayed (cutoff *p*-value = 0.01). (PDF 45 kb) Additional file 7:
**Number of genes in common between GES.** (PDF 46 kb) Additional file 8:
**Number of GO enrichment terms (biological tree) in common between GES’s gene lists.** (PDF 68 kb) Additional file 9:
**Percentage of GO enrichment terms (biological tree) in common between GES’s gene lists, sorted in decreasing order.** (PDF 99 kb)

## Abbreviations

70-GES: van’t Veer prognostic GES
AG1: age group n°1 (≤40 years)
AG2: age group n°2 (>40 to < 70 years)
AG3: age group n°3 (≥70 years)
ANOVA: analysis of variance
CIN: chromosomal instability
CSC: cancer stem-like cells
ER: oestrogen receptor (+: positive)
GES: gene-expression signature
GGI: genomic grade index
IFN: interferon
IHC: immunohistochemistry
IR: immune response
IRGS: iron regulatory gene signature
IL-8: interleukin-8
MITO/OXPHOS: mitochondrial oxidative phosphorylation; M2-macrophages/M1-macrophages enrichment
N: nodal status (+: positive)
OR: odds ratio
PAM: prediction analysis for microarrays
RS: recurrence score
SAM: significance analysis of microarrays
SBR: Scarff-Bloom-Richardson histological grade
TNBC: triple negative breast cancer
UMLR: unordered multinomial logistic regression
VEGF: vascular endothelial growth factor
