# Supplementary material for: Gene-expression signature functional annotation of breast cancer tumours in function of age
Source: BMC Med Genomics. 2015 Nov 23;8:80. doi: 10.1186/s12920-015-0153-6 (PMC4657228; doi:10.1186/s12920-015-0153-6)
Supplement: Additional file 1: — GES listing, methods and references. (PDF 114 kb) [file 12920_2015_153_MOESM1_ESM.pdf]

Additional file 1: GES details. 1A: GES list, methods and references; **1B**: Number of GES genes or probes present in each array type.

1A

N°	GES name	n genes	n probes	Normalisation	Statistics	Reference
**Molecular subtyping**						
1	PAM50	50	-	MAS5/log2 transformation	Nearest centroid classifier	[1]
2	ER	135	-	RMA	Weighted average expression	[2]
3	Molecular-apocrine	-	27	MAS5/log2 transformation	Average expression	[3]
4	Basal-like	-	37	MAS5/log2 transformation	Average expression	[3]
5	Claudin-CD24	-	19	MAS5/log2 transformation	Average expression	[3]
**Immune response**						
6	B-cell	-	48	MAS5/log2 transformation	Average expression	[3]
7	T-Cell	-	27	MAS5/log2 transformation	Average expression	[3]
8	MHC-1	-	17	MAS5/log2 transformation	Average expression	[3]
9	MHC-2	-	14	MAS5/log2 transformation	Average expression	[3]
10	M2/M1	611	-	MAS5/log2 transformation	Weighted average expression	[4]
11	IFN	-	14	MAS5/log2 transformation	Average expression	[3]
12	IL-8	-	4	MAS5/log2 transformation	Average expression	[3]
**Metabolism**						
13	Adipocytes	-	8	MAS5/log2 transformation	Average expression	[3]
14	Glycolysis	6	-	MAS5/log2 transformation	Average expression	[5]
15	IRGS	-	19	RMA	Weighted average expression	[6]
**Critical biological pathways in cancer**						
16	CIN	25	-	RMA	Sum expression	[7]
17	ERBB2	4	-	RMA	Weighted average expression	[2]
18	HOXA	-	8	MAS5/log2 transformation	Average expression	[3]
19	MITO/OXPHOS	38	-	MAS5/log2 transformation	average expression	[8
20	Proliferation	-	47	MAS5/log2 transformation	Average expression	[3]
21	Reactive stroma	-	47	MAS5/log2 transformation	Average expression	[3]
22	VEGF	-	7	MAS5/log2 transformation	Average expression	[3]
**Prognosis**						
23	70-GES	70	-	MAS5/log2 transformation	Nearest centroid classifier	[9]
24	GGI	-	128	RMA	Weighted average expression	[10]
25	Recurrence score	21	-	MAS5/log2 transformation	Recurrence score algorithm	[11]

1B

N°	GES name	n genes	n probes	HG-U133A		HG-U133A + B		HG-U133Plus2		HG-U95A	
				n	%	n	%	n	%	n	%
**Molecular subtyping**											
1	PAM50	50	-	43	86	49	98	49	98	39	78
2	ER	135	-	127	94	127	94	127	94	102	76
3	Molecular-apocrine	-	27	27	100	27	100	27	100	0	0
4	Basal-like	-	37	37	100	37	100	37	100	1	3
5	Claudin-CD24	-	19	19	100	19	100	19	100	2	11
**Immune response**											
6	B-cell	-	48	48	100	48	100	48	100	0	0
7	T-Cell	-	27	27	100	27	100	27	100	2	7
8	MHC-1	-	17	17	100	17	100	17	100	0	0
9	MHC-2	-	14	14	100	14	100	14	100	0	0
10	M2/M1	611	-	482	79	597	98	607	99	374	61
11	IFN	-	14	14	100	14	100	14	100	0	0
12	IL-8	-	4	4	100	4	100	4	100	0	0
**Metabolism**											
13	Adipocytes	-	8	8	100	8	100	8	100	0	0
14	Glycolysis	6	-	5	83	5	83	5	83	5	83
15	IRGS	-	19	9	47	19	100	19	100	0	0
**Critical biological pathways in cancer**											
16	CIN	25	-	25	100	25	100	25	100	23	92
17	ERBB2	4	-	4	100	4	100	4	100	3	75
18	HOXA	-	8	8	100	8	100	8	100	0	0
19	MITO/OXPHOS	38	-	36	95	36	95	36	95	30	79
20	Proliferation	-	47	47	100	47	100	47	100	0	0
21	Reactive stroma	-	47	47	100	47	100	47	100	0	0
22	VEGF	-	7	7	100	7	100	7	100	0	0
**Prognosis**											
23	70-GES	70	-	50	77	59	91	60	92	33	51
24	GGI	-	128	128	100	128	100	128	100	1	1
25	Recurrence score	21	-	16	89	18	100	18	100	0	0

References

[1] Sorlie T, Tibshirani R, Parker J, Hasties T, Marron JS, Nobel A, Deng S, Johnsen H, Pesich R, Geisler S, Demeter J, Perou CM, Lonning PE, Brown PO, Borresen-Dale AL, Botstein D: **Repeated observation of breast tumor subtypes in independent gene expression data sets.** *Proc Natl Acad Sci USA* 2003, **100:**8418-8423.

[2] Wolf DM, Lenburg ME, Yau C, Boudreau A, van't Veer LJ. **Gene co-expression modules as clinically relevant hallmarks of breast cancer diversity.** *PLoS One* 2014, **9:**e88309.

[3] Karn T, Pusztai L, Holtrich U, Iwamoto T, Shiang CY, Schmidt M, Müller V, Solbach C, Gaetje R, Hanker L, Ahr A, Liedtke C, Ruckhäberle E, Kaufmann M, Rody A. **Homogeneous datasets of triple negative breast cancers enable the identification of novel prognostic and predictive signatures.** *PLoS One* 2011, **6:**e28403.

[4] Jézéquel P, Loussouarn D, Guérin-Charbonnel C, Campion L, Vanier A, Gouraud W, Lasla H, Guette C, Valo I, Verrièle V, Campone M. **Gene-expression molecular subtyping of triple-negative breast cancer tumours: importance of immune response.** *Breast Cancer Res* 2015, **17:**43.

[5] Hu Z, Fan C, Livasy C, He X, Oh DS, Ewend MG, Carey LA, Subramanian S, West R, Ikpatt F, Olopade OI, van de Rijn M, Perou CM: **A compact VEGF signature associated with distant metastases and poor outcomes.** *BMC Med* 2009, **7:**9.

[6] Miller LD, Coffman LG, Chou JW, Black MA, Bergh J, D'Agostino R Jr, Torti SV, Torti FM: **An Iron Regulatory Gene Signature Predicts Outcome in Breast Cancer.** *Cancer Res* 2011, **71:**6728-6737.

[7] Carter SL, Eklund AC, Kohane IS, Harris LN, Szallasi Z: **A signature of chromosomal instability inferred from gene expression profiles predicts clinical outcome in multiple human cancers.** *Nat Genet* 2006, **9:**1043-1048.

[8] Whitaker-Menezes D, Martinez-Outschoorn UE, Flomenberg N, Birbe RC, Witkiewicz AK, Howell A, Pavlides S, Tsirigos A, Ertel A, Pestell RG, Broda P, Minetti C, Lisanti MP, Sotgia F: **Hyperactivation of oxidative mitochondrial metabolism in epithelial cancer cells in situ: visualizing the therapeutic effects of metformin in tumor tissue.** *Cell Cycle* 2011, **23:**4047-4064.

[9] van 't Veer LJ, Dai H, van de Vijver MJ, He YD, Hart AA, Mao M, Peterse HL, van der Kooy K, Marton MJ, Witteveen AT, Schreiber GJ, Kerkhoven RM, Roberts C, Linsley PS, Bernards R, Friend SH: **Gene expression profiling predicts clinical outcome of breast cancer**. *Nature* 2002, **415:**530-536.

[10] Sotiriou C, Wirapati P, Loi S, Harris A, Fox S, Smeds J, Nordgren H, Farmer P, Praz V, Haibe-Kains B, Desmedt C, Larsimont D, Cardoso F, Peterse H, Nuyten D, Buyse M, Van de Vijver MJ, Bergh J, Piccart M, Delorenzi M: **Gene expression profiling in breast cancer: understanding the molecular basis of histologic grade to improve prognosis.** *J Natl Cancer Inst* 2006, **98:**262–272.

[11] Paik S, Shak S, Tang G, Kim C, Baker J, Cronin M, Baehner FL, Walker MG, Watson D, Park T, Hiller W, Fisher ER, Wickerham DL, Bryant J, Wolmark N: **A multigene assay to predict recurrence of tamoxifentreated node-negative breast cancer**. *N Engl J Med* 2004, **351:**2817-2826.
