# Supplementary material for: Gene-expression signature functional annotation of breast cancer tumours in function of age
Source: BMC Med Genomics. 2015 Nov 23;8:80. doi: 10.1186/s12920-015-0153-6 (PMC4657228; doi:10.1186/s12920-015-0153-6)

Additional file 2: Metastasis-free survival (MFS) and overall survival (OS) analyses. MFS: 2A1: whole cohort; 2A2: Caucasian cohort. OS: 2B1: whole cohort; 2B2: Caucasian cohort.

2A1

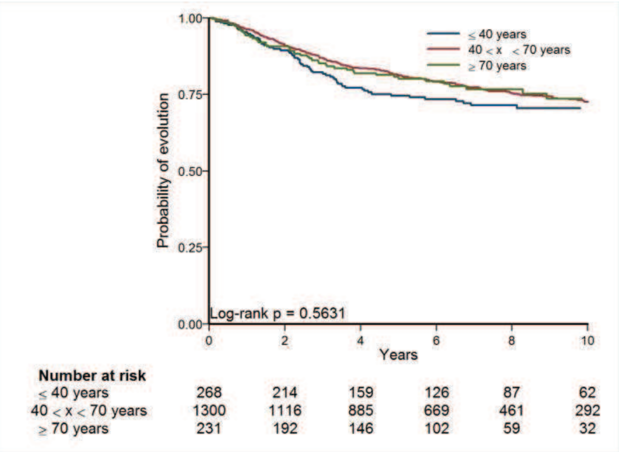

2A2

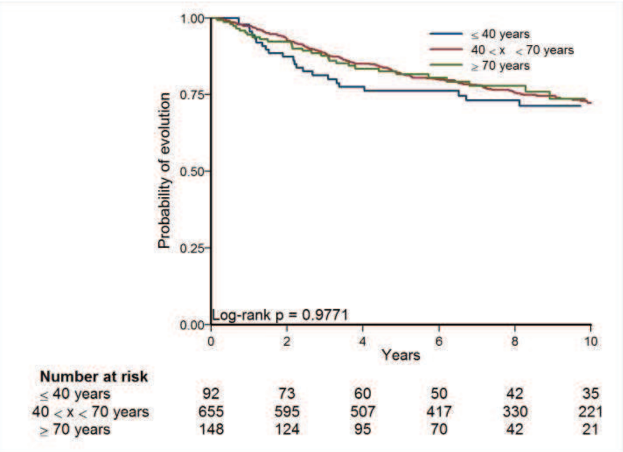

2B1

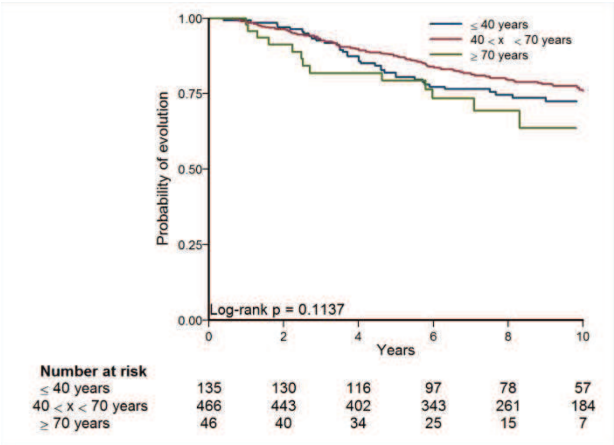

2B2

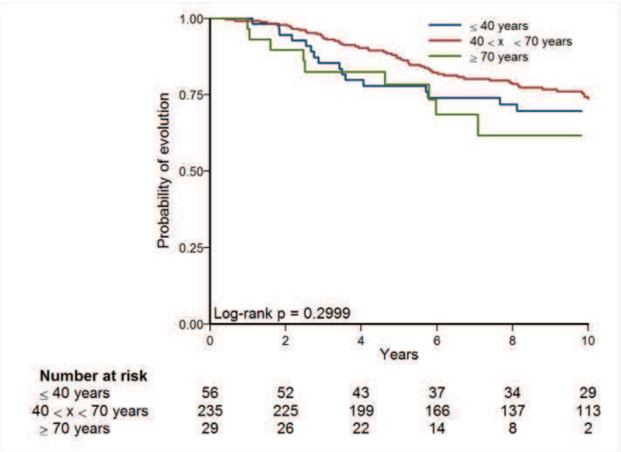

Supplement: Additional file 2: — Metastasis-free survival (MFS) and overall survival (OS) analyses. MFS: 2A1: whole cohort; 2A2: Caucasian cohort. OS: 2B1: whole cohort; 2B2: Caucasian cohort. (PDF 230 kb) [file 12920_2015_153_MOESM2_ESM.pdf]
