# Supplementary material for: Gene-expression signature functional annotation of breast cancer tumours in function of age
Source: BMC Med Genomics. 2015 Nov 23;8:80. doi: 10.1186/s12920-015-0153-6 (PMC4657228; doi:10.1186/s12920-015-0153-6)
Supplement: Additional file 3: — GES scoring of breast cancer in function of age. Correlation matrices show Pearson correlation coefficients between continuous GES (red indicates a positive correlation; blue, a negative correlation) and dendrograms show mutual relationships of all signatures (scores were used for average-link hierarchical clustering using the Pearson correlation as a distance metric). 2A1: AG1, whole cohort; 2A2: AG2, whole cohort; 2A3: AG3 whole cohort; 2B1: AG1, Caucasian cohort; 2B2: AG2, Caucasian cohort; 2B3: AG3, Caucasian cohort. (PDF 1689 kb) [file 12920_2015_153_MOESM3_ESM.pdf]

Additional file 3: GES scoring of breast cancer in function of age. Correlation matrices show Pearson correlation coefficients between continuous GES (red indicates a positive correlation; blue, a negative correlation) and dendrograms show mutual relationships of all signatures (scores were used for average-link hierarchical clustering using the Pearson correlation as a distance metric). **3A1**: AG1, whole cohort; **3A2**: AG2, whole cohort; **3A3**: AG3 whole cohort; **3B1**: AG1, Caucasian cohort; **3B2**: AG2, Caucasian cohort; **3B3**: AG3, Caucasian cohort.

3A1

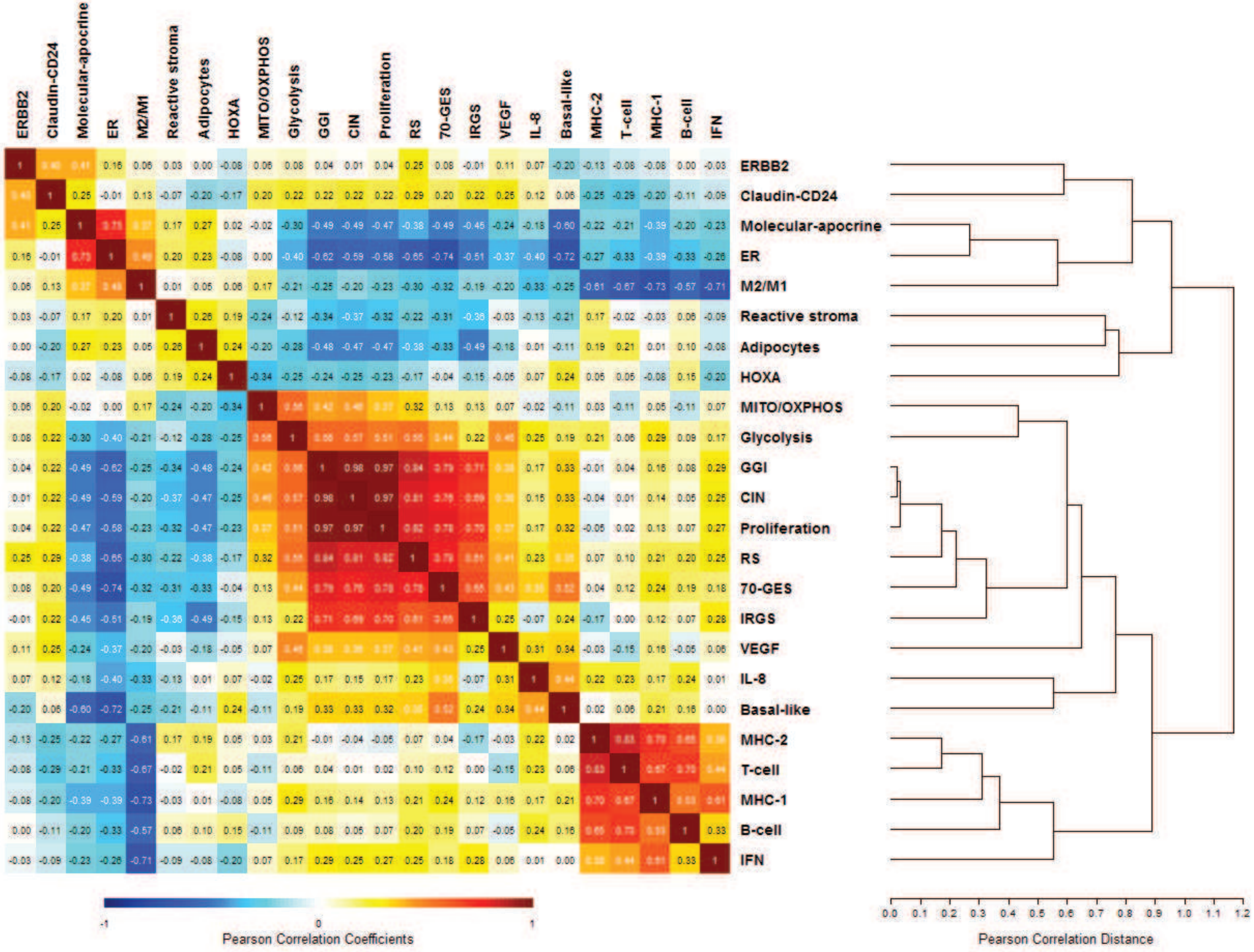
ERBB2
Claudin-CD24
Molecular-apocrine
ER
M2/M1
Reactive stroma
Adipocytes
HOXA
MITO/OXPHOS
Glycolysis
GGI
CIN
Proliferation
RS
70-GES
IRGS
VEGF
IL-8
Basal-like
MHC-2
T-cell
MHC-1
B-cell
IFN
-1
0
1
Pearson Correlation Coefficients
0.0
0.1
0.2
0.3
0.4
0.5
0.6
0.7
0.8
0.9
1.0
1.1
1.2
Pearson Correlation Distance

3A2

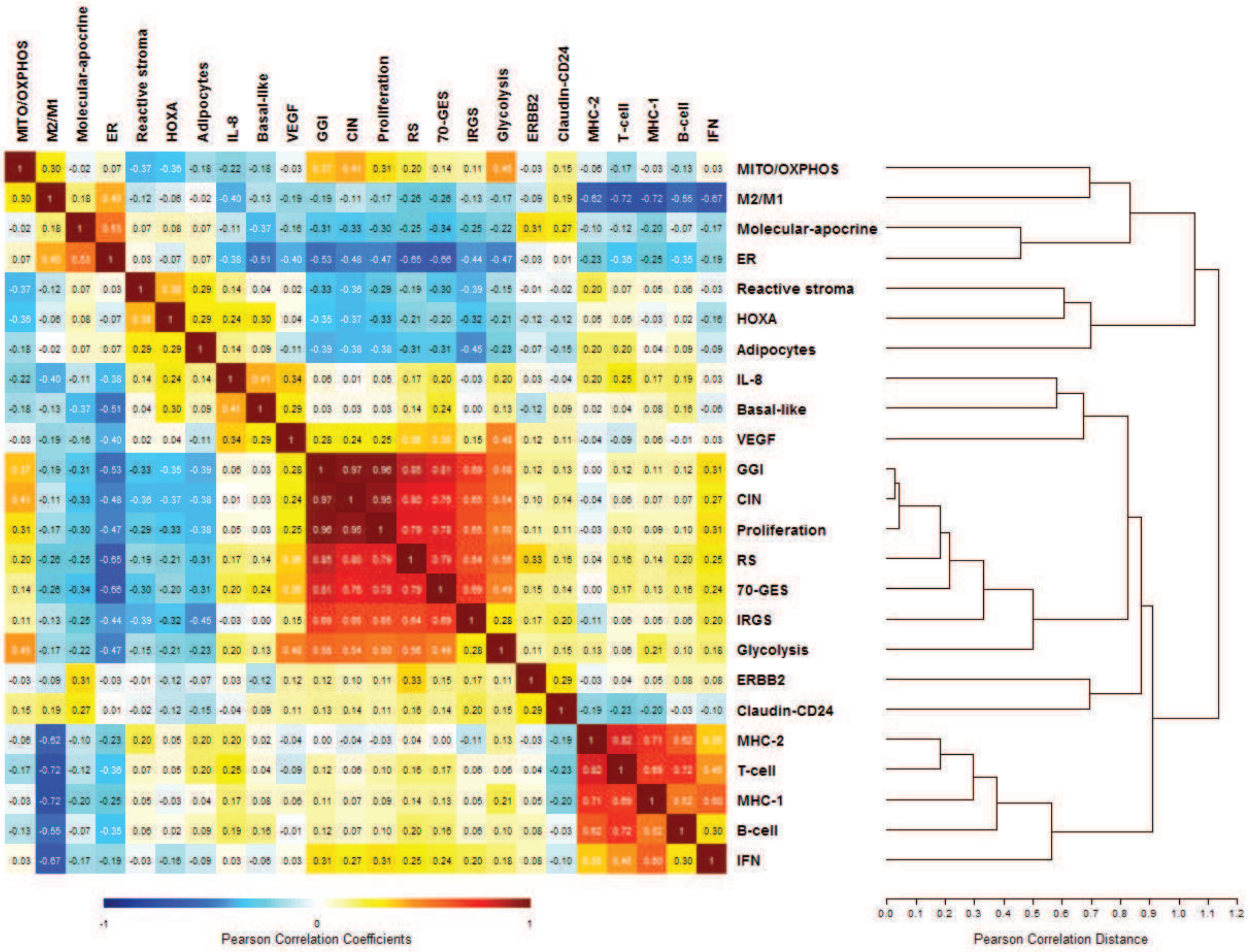

MITO/OXPHOS
M2/M1
Molecular-apocrine
ER
Reactive stroma
HOXA
Adipocytes
IL-8
Basal-like
VEGF
GGI
CIN
Proliferation
RS
70-GES
IRGS
Glycolysis
ERBB2
Claudin-CD24
MHC-2
T-cell
MHC-1
B-cell
IFN
-1
0
1
Pearson Correlation Coefficients
0.0 0.1 0.2 0.3 0.4 0.5 0.6 0.7 0.8 0.9 1.0 1.1 1.2
Pearson Correlation Distance

3A3

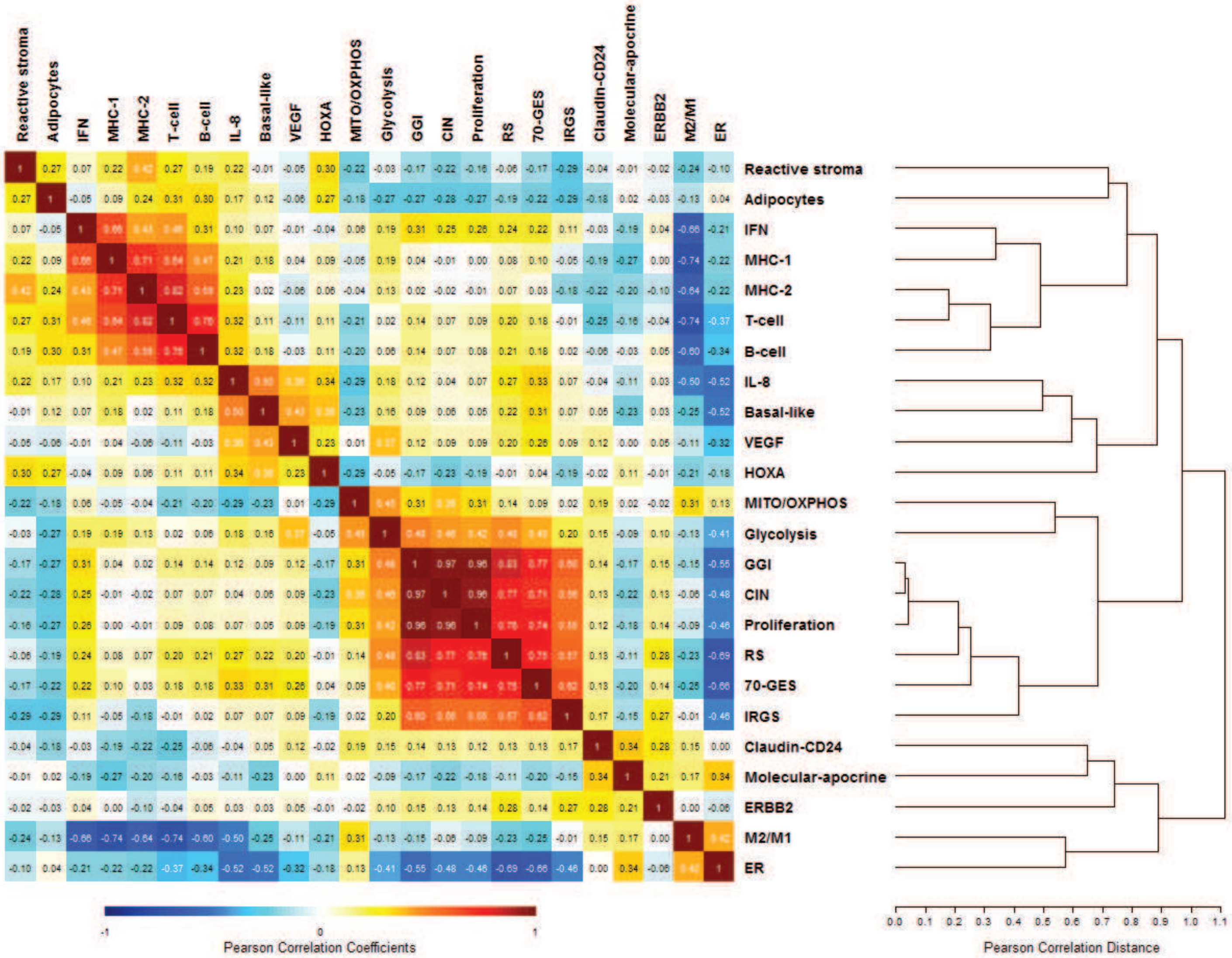

Reactive stroma
Adipocytes
IFN
MHC-1
MHC-2
T-cell
B-cell
IL-8
Basal-like
VEGF
HOXA
MITO/OXPHOS
Glycolysis
GGI
CIN
Proliferation
RS
70-GES
IRGS
Claudin-CD24
Molecular-apocrine
ERBB2
M2/M1
ER
-1
0
1
Pearson Correlation Coefficients
0.0
0.1
0.2
0.3
0.4
0.5
0.6
0.7
0.8
0.9
1.0
1.1
Pearson Correlation Distance

3B1

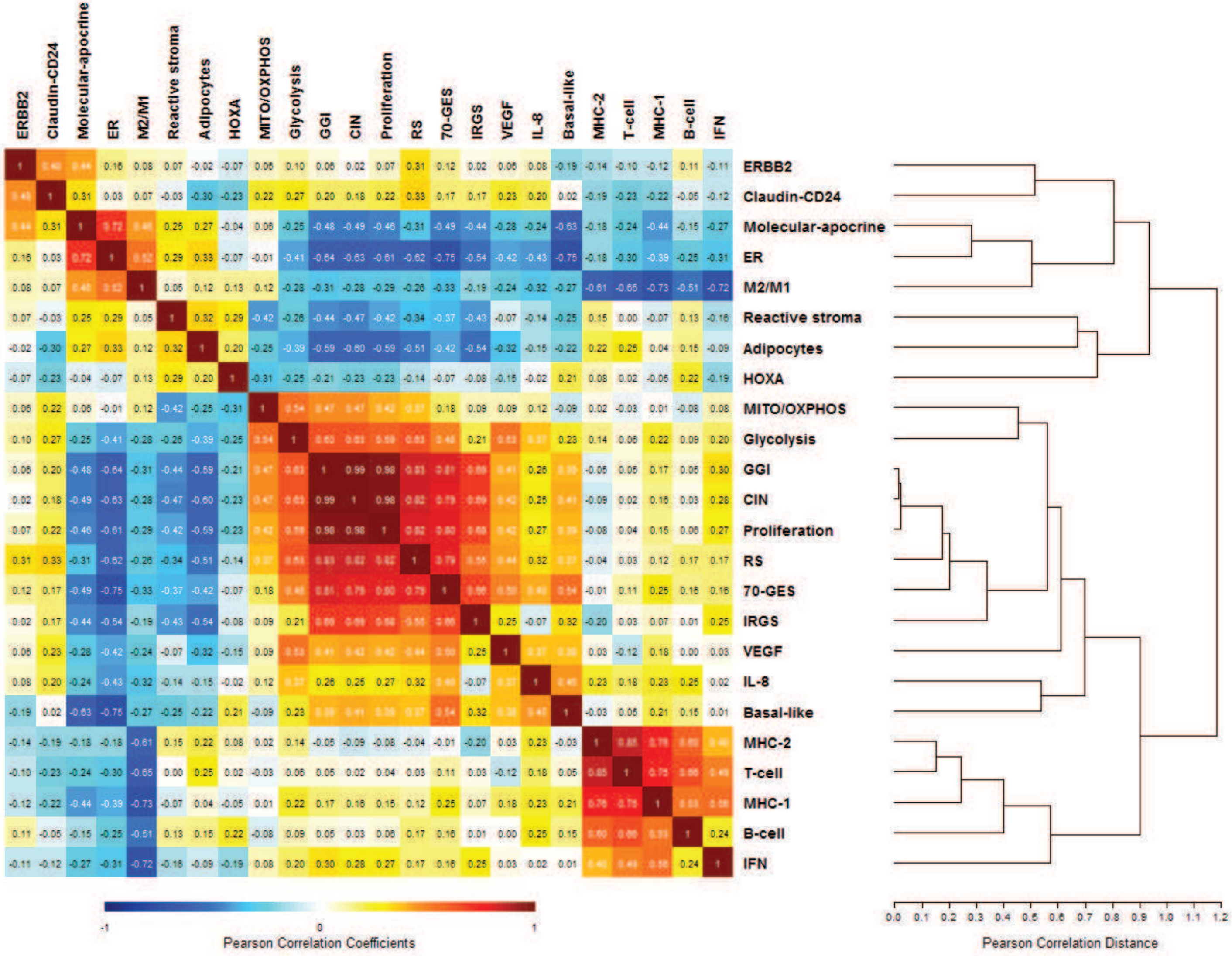
ERBB2
Claudin-CD24
Molecular-apocrine
ER
M2/M1
Reactive stroma
Adipocytes
HOXA
MITO/OXPHOS
Glycolysis
GGI
CIN
Proliferation
RS
70-GES
IRGS
VEGF
IL-8
Basal-like
MHC-2
T-cell
MHC-1
B-cell
IFN
-1
0
1
Pearson Correlation Coefficients
0.0 0.1 0.2 0.3 0.4 0.5 0.6 0.7 0.8 0.9 1.0 1.1 1.2
Pearson Correlation Distance

3B2

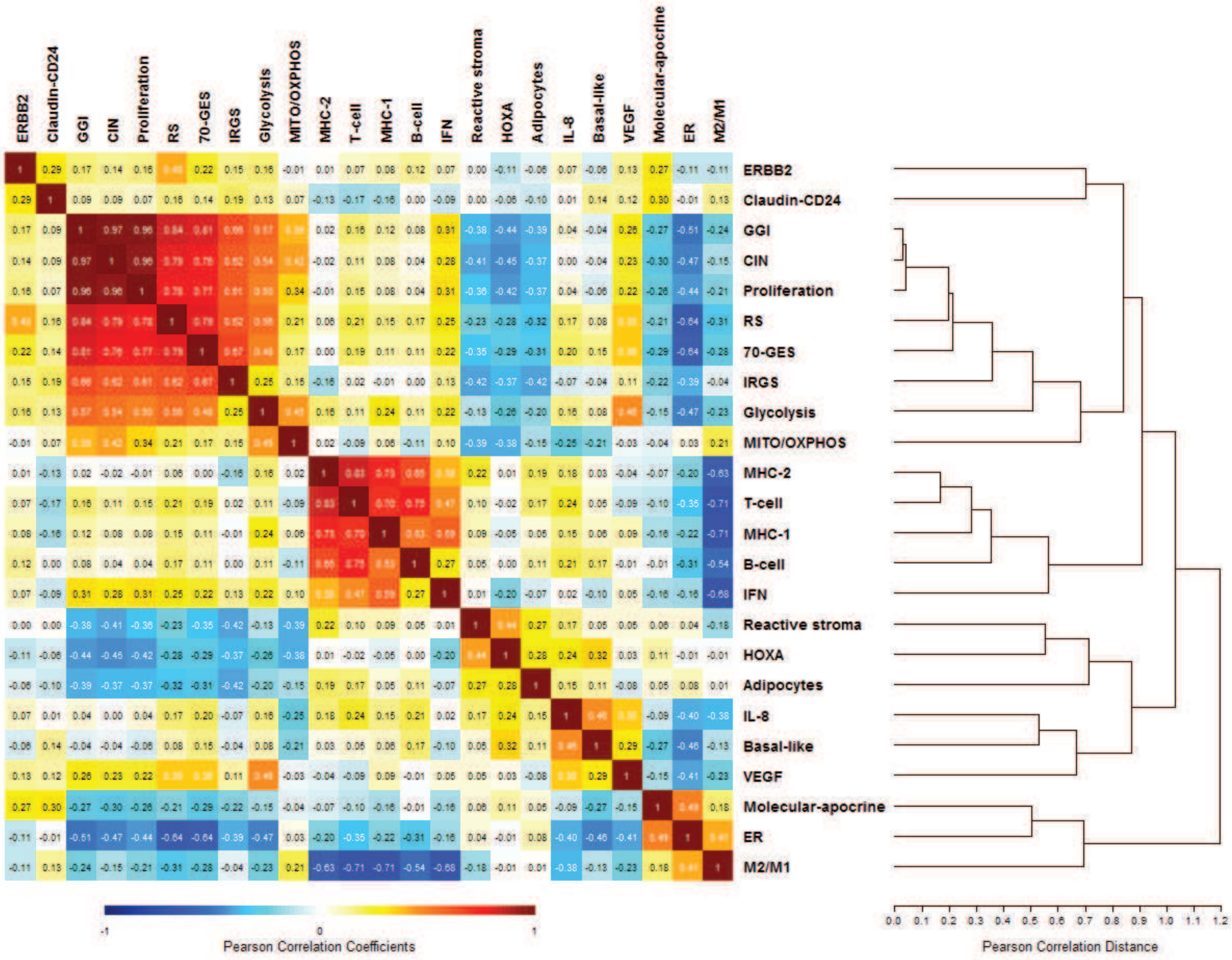

ERBB2
Claudin-CD24
GGI
CIN
Proliferation
RS
70-GES
IRGS
Glycolysis
MITO/OXPHOS
MHC-2
T-cell
MHC-1
B-cell
IFN
Reactive stroma
HOXA
Adipocytes
IL-8
Basal-like
VEGF
Molecular-apocrine
ER
M2/M1
-1
0
1
Pearson Correlation Coefficients
0.0 0.1 0.2 0.3 0.4 0.5 0.6 0.7 0.8 0.9 1.0 1.1 1.2
Pearson Correlation Distance

3B3

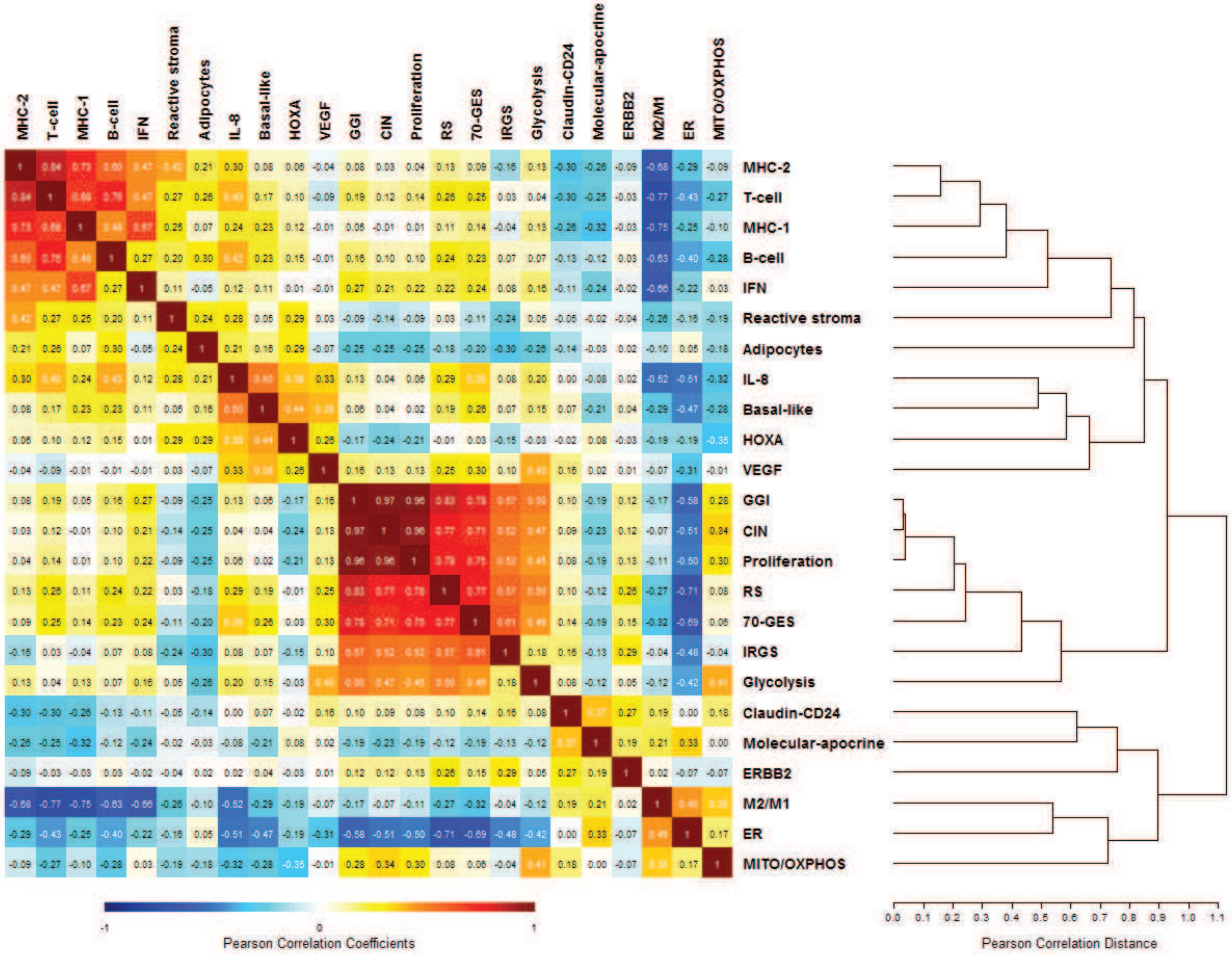
MHC-2
T-cell
MHC-1
B-cell
IFN
Reactive stroma
Adipocytes
IL-8
Basal-like
HOXA
VEGF
GGI
CIN
Proliferation
RS
70-GES
IRGS
Glycolysis
Claudin-CD24
Molecular-apocrine
ERBB2
M2/M1
ER
MITO/OXPHOS
-1
0
1
Pearson Correlation Coefficients
0.0 0.1 0.2 0.3 0.4 0.5 0.6 0.7 0.8 0.9 1.0 1.1
Pearson Correlation Distance
