# Supplementary material for: Gene-expression signature functional annotation of breast cancer tumours in function of age
Source: BMC Med Genomics. 2015 Nov 23;8:80. doi: 10.1186/s12920-015-0153-6 (PMC4657228; doi:10.1186/s12920-015-0153-6)
Supplement: Additional file 4: — GES analyses differentiating AG1 and AG3 luminal B patients. AG1 > AG3 means that AG1 GES score is superior to AG3 GES score, and inversely. (PDF 57 kb) [file 12920_2015_153_MOESM4_ESM.pdf]

Additional file 4: GES analyses differentiating AG1 and AG3 luminal B patients. AG1 > AG3 means that AG1 GES score is superior to AG3 GES score, and inversely.

GES name	Whole cohort		Caucasian cohort	
	p-value	Results	p-value	Results
**Metabolism**				
IRGS	0.0008	AG1 > AG3	0.2130	NS
**Critical biological pathways in cancer**				
MITO/OXPHOS	0.0035	AG1 < AG3	0.0563	NS
Reactive stroma	0.0118	AG1 > AG3	0.0010	AG1 > AG3
**Prognosis**				
RS	0.0005	AG1 > AG3	0.0089	AG1 > AG3

AG1: age group 1 (≤ 40 years); AG3: age group 2 (≥ 70 years); NS: not significant
