# Supplementary material for: Gene-expression signature functional annotation of breast cancer tumours in function of age
Source: BMC Med Genomics. 2015 Nov 23;8:80. doi: 10.1186/s12920-015-0153-6 (PMC4657228; doi:10.1186/s12920-015-0153-6)
Supplement: Additional file 5: — Continuous GES analyses interpretation in function of ER status in the three age groups. (PDF 75 kb) [file 12920_2015_153_MOESM5_ESM.pdf]

Additional file 5: Continuous GES analyses interpretation in function of ER status in the three age groups.

GES name	ER + whole cohort						ER- whole cohort					
	p-value	p- value			Results		p- value	p- value			Results	
		1 vs 2	1 vs 3	2 vs 3				1 vs 2	1 vs 3	2 vs 3		
**Molecular subtyping**												
ER	0.4895				NS		0.0294	0.0707	0.0479	0.5066	1 < 3	1 ≈ 2 and 2 ≈ 3
Molecular-apocrine	0.0122	0.0097	0.1878	0.5649	1 < 2	1 ≈ 3 and 2 ≈ 3	< 0.0001	0.0003	0.0002	0.1961	1 < 2 ≈ 3	
Basal-like	0.0016	0.3550	0.0029	0.0070	1 ≈ 2 > 3		< 0.0001	< 0.0001	0.0018	0.7710	1 > 2 ≈ 3	
Claudin-CD24	0.6379				NS		0.8944				NS	
**Immune response**												
B-cell	0.0043	0.8412	0.1513	0.0028	2 > 3	1 ≈ 2 and 1 ≈ 3	0.0048	0.9830	0.0085	0.0040	1 ≈ 2 > 3	
T-cell	0.0204	0.1610	0.9873	0.0436	2 > 3	1 ≈ 2 and 1 ≈ 3	0.2050				NS	
MHC-1	0.1260				NS		0.0469	0.2932	0.0388	0.2024	1 > 3	1 ≈ 2 and 2 ≈ 3
MHC-2	0.0007	0.0006	0.1021	0.2697	1 < 2	1 ≈ 3 and 2 ≈ 3	0.4598				NS	
M2/M1	0.7480				NS		0.3799				NS	
IFN	0.0950				NS		0.4597				NS	
IL-8	0.4711				NS		0.1422				NS	
**Metabolism**												
Adipocytes	0.0195	0.0370	0.6829	0.1828	1 < 2	1 ≈ 3 and 2 ≈ 3	0.5114				NS	
Glycolysis	0.6257				NS		0.6687				NS	
IRGS	0.0360	0.0302	0.2271	0.6459	1 > 2	1 ≈ 3 and 2 ≈ 3	0.7623				NS	
**Critical biological pathways in cancer**												
CIN	0.0066	0.0148	0.6121	0.1191	1 > 2	1 ≈ 3 and 2 ≈ 3	0.0959				NS	
ERBB2	0.1921				NS		0.0827				NS	
HOXA	0.2943				NS		0.0056	0.4192	0.1041	0.0050	2 < 3	1 ≈ 2 and 1 ≈ 3
MITO/OXPHOS	0.0002	0.0733	0.0002	0.0057	1 ≈ 2 < 3		0.0903				NS	
Proliferation	0.0018	0.0027	0.3099	0.1429	1 > 2	1 ≈ 3 and 2 ≈ 3	0.3159				NS	
Reactive stroma	< 0.0001	0.5789	0.0097	< 0.0001	1 ≈ 2 > 3		0.5584				NS	
VEGF	0.5569				NS		0.5087				NS	
**Prognosis**												
70-GES	0.0097	0.0680	0.9882	0.0386	2 > 3	1 ≈ 2 and 1 ≈ 3	0.1505				NS	
GGI	0.0173	0.0278	0.5861	0.2177	1 > 2	1 ≈ 3 and 2 ≈ 3	0.5285				NS	
RS	0.0023	0.0082	0.0019	0.4079	1 > 2 ≈ 3		0.9230				NS	

ER: estrogen receptor; 1: ≤ 40 years (ER+, n = 199; ER-, n = 143); 2: 40 to < 70 years (ER+, n = 1170; ER-, n = 477); 3: ≥ 70 years (ER+, n = 302; ER-, n = 61); IFN: interferon; IRGS: iron regulatory gene signature; CIN: chromosomal instability; MITO/OXPHOS: mitochondrial oxidative phosphorylation; NS: not significant (p > 0.05); 70-GES: van't Veer et al prognostic GES; GGI: genomic grade index; RS: recurrence score
