# Supplementary material for: Gene-expression signature functional annotation of breast cancer tumours in function of age
Source: BMC Med Genomics. 2015 Nov 23;8:80. doi: 10.1186/s12920-015-0153-6 (PMC4657228; doi:10.1186/s12920-015-0153-6)
Supplement: Additional file 6: — GO biological process enrichment analyses of most differentially expressed genes between AG1 and AG3. Head of columns represents SAM gene lists used as ToppGene inputs. The ten first significant biological processes are displayed (cutoff p-value = 0.01). (PDF 45 kb) [file 12920_2015_153_MOESM6_ESM.pdf]

Additional file 6: GO biological process enrichment analyses of most differentially expressed genes between AG1 and AG3. Head of columns represents SAM gene lists used as ToppGene inputs. The ten first significant biological processes are displayed (cutoff p-value = 0.01).

	**Genes overexpressed in AG1 patients versus AG3 patients, (n = 677 out of 684)**	**Genes overexpressed in AG3 patients versus AG1 patients, (n = 428 out of 432)**
1	Cell proliferation	Oxidation-reduction process
2	Movement of cell or subcellular component	Carboxylic acid catabolic process
3	Mitotic cell cycle	Organic acid catabolic process
4	Regulation of cell proliferation	Small molecule catabolic process
5	Mitotic cell cycle process	Oxoacid metabolic process
6	Cell cycle process	Organic acid metabolic process
7	Cell migration	Lipid metabolic process
8	Localization of cell	Carboxylic acid metabolic process
9	Cell motility	Cellular lipid metabolic process
10	Positive regulation of cell proliferation	Fatty acid beta-oxydation
