# Supplementary material for: Gene-expression signature functional annotation of breast cancer tumours in function of age
Source: BMC Med Genomics. 2015 Nov 23;8:80. doi: 10.1186/s12920-015-0153-6 (PMC4657228; doi:10.1186/s12920-015-0153-6)
Supplement: Additional file 7: — Number of genes in common between GES. (PDF 46 kb) [file 12920_2015_153_MOESM7_ESM.pdf]

Additional file 7: Number of genes in common between GES.

	ER	Molecular-apocrine	Basal-like	Claudin-CD24	B-Cell	T-Cell	MHC-1	MHC-2	M2/M1	IFN	IL-8	Adipocytes	Glycolysis	IRGS	CIN	ERBB2	HOXA	MITO/ OXPHOS	Proliferation	Reactive stroma	VEGF	70-GES	GGI	RS	PAM50
**ER**	135	9	0	0	0	0	0	0	6	0	0	0	0	0	0	0	0	0	0	0	0	1	0	3	6
**Molecular-apocrine**	9	16	0	0	0	0	0	0	1	0	0	0	0	0	0	0	0	0	0	0	0	0	0	0	2
**Basal-like**	0	0	25	0	0	0	0	0	0	0	0	0	0	0	0	0	0	0	0	0	0	0	0	0	5
**Claudin-CD24**	0	0	0	11	0	0	0	0	0	0	0	0	0	0	0	0	0	0	0	0	0	0	0	0	0
**B-Cell**	0	0	0	0	21	0	0	0	0	0	0	0	0	0	0	0	0	0	0	0	0	0	0	0	0
**T-Cell**	0	0	0	0	0	21	0	0	3	0	0	0	0	0	0	0	0	0	0	0	0	0	0	0	0
**MHC-1**	0	0	0	0	0	0	7	0	2	0	0	0	0	0	0	0	0	0	0	0	0	0	0	0	0
**MHC-2**	0	0	0	0	0	0	0	9	0	0	0	0	0	0	0	0	0	0	0	0	0	0	0	0	0
**M2/M1**	6	1	0	0	0	3	2	0	611	5	2	1	0	3	0	0	0	2	1	1	0	1	3	0	3
**IFN**	0	0	0	0	0	0	0	0	5	13	0	0	0	0	0	0	0	0	0	0	0	0	0	0	0
**IL-8**	0	0	0	0	0	0	0	0	2	0	3	0	0	0	0	0	0	0	0	0	0	0	0	0	0
**Adipocytes**	0	0	0	0	0	0	0	0	1	0	0	6	0	0	0	0	0	0	0	0	0	0	0	0	0
**Glycolysis**	0	0	0	0	0	0	0	0	0	0	0	0	6	0	0	0	0	0	0	0	0	0	0	1	0
**IRGS**	0	0	0	0	0	0	0	0	3	0	0	0	0	16	0	0	0	0	0	0	0	0	1	1	0
**CIN**	0	0	0	0	0	0	0	0	0	0	0	0	0	0	25	0	0	0	10	0	0	3	17	0	2
**ERBB2**	0	0	0	0	0	0	0	0	0	0	0	0	0	0	0	4	0	0	0	0	0	0	0	2	2
**HOXA**	0	0	0	0	0	0	0	0	0	0	0	0	0	0	0	0	6	0	0	0	0	0	0	0	0
**MITO/ OXPHOS**	0	0	0	0	0	0	0	0	2	0	0	0	0	0	0	0	0	37	0	0	0	0	0	0	0
**Proliferation**	0	0	0	0	0	0	0	0	1	0	0	0	0	0	10	0	0	0	40	0	0	4	33	3	7
**Reactive stroma**	0	0	0	0	0	0	0	0	1	0	0	0	0	0	0	0	0	0	0	26	0	0	0	0	0
**VEGF**	0	0	0	0	0	0	0	0	0	0	0	0	0	0	0	0	0	0	0	0	4	0	0	0	0
**70-GES**	1	0	0	0	0	0	0	0	1	0	0	0	0	0	3	0	0	0	4	0	0	61	8	1	3
**GGI**	0	0	0	0	0	0	0	0	3	0	0	0	0	1	17	0	0	0	33	0	0	8	110	5	14
**RS**	3	0	0	0	0	0	0	0	0	0	0	0	1	1	0	2	0	0	3	0	0	1	5	21	11
**PAM50**	6	2	5	0	0	0	0	0	3	0	0	0	0	0	2	2	0	0	7	0	0	3	14	11	50
