# Supplementary material for: Gene-expression signature functional annotation of breast cancer tumours in function of age
Source: BMC Med Genomics. 2015 Nov 23;8:80. doi: 10.1186/s12920-015-0153-6 (PMC4657228; doi:10.1186/s12920-015-0153-6)
Supplement: Additional file 8: — Number of GO enrichment terms (biological tree) in common between GES’s gene lists. (PDF 68 kb) [file 12920_2015_153_MOESM8_ESM.pdf]

Additional file 8: Number of GO enrichment terms (biological tree) in common between GES's gene lists.

overlap per column	ER	Molecular-apocrine	Basal-like	Claudin-CD24	B-Cell	T-Cell	MHC-1	MHC-2	M2/M1	IFN	IL-8	Adipocytes	Glycolysis	IRGS	CIN	ERBB2	HOXA	MITO/ OXPHOS	Proliferation	Reactive stroma	VEGF	70-GES	GGI	RS	PAM50
**ER**	100.0	17.5	0.0	0.0	0.0	0.0	0.0	2.1	1.6	0.0	0.0	0.0	0.0	0.0	0.0	0.0	7.0	0.0	1.1	1.2	1.8	0.0	0.0	14.0	11.0
**Molecular-apocrine**	15.2	100.0	0.0	2.0	0.0	2.1	0.0	0.0	0.8	0.0	0.0	1.2	0.0	0.0	0.0	0.0	4.7	0.0	0.0	0.0	0.0	0.0	0.0	1.0	1.7
**Basal-like**	0.0	0.0	100.0	2.0	0.0	0.0	0.0	0.0	0.0	0.0	0.0	0.0	0.0	0.0	1.5	0.0	0.0	0.0	0.0	0.0	0.0	0.0	0.0	0.0	9.8
**Claudin-CD24**	0.0	1.8	2.1	100.0	0.0	2.1	3.4	0.0	0.8	0.0	16.7	0.0	0.0	0.0	0.0	0.0	0.0	0.0	0.0	0.0	0.9	0.0	0.0	0.0	0.0
**B-Cell**	0.0	0.0	0.0	0.0	100.0	2.1	10.3	2.1	1.6	3.3	16.7	0.0	0.0	0.0	0.0	0.0	0.0	0.0	0.0	0.0	0.0	0.0	0.0	0.0	0.0
**T-Cell**	0.0	1.8	0.0	2.0	8.3	100.0	3.4	8.3	0.8	0.0	0.0	0.0	0.0	0.0	0.0	0.0	0.0	2.8	0.0	2.4	0.0	0.0	1.0	0.0	0.0
**MHC-1**	0.0	0.0	0.0	2.0	25.0	2.1	100.0	10.4	6.3	13.3	16.7	1.2	0.0	0.0	0.0	0.0	0.0	0.0	0.0	0.0	0.0	0.0	0.0	0.0	0.0
**MHC-2**	1.5	0.0	0.0	0.0	8.3	8.5	17.2	100.0	4.8	10.0	16.7	1.2	0.0	0.0	0.0	0.0	0.0	0.0	1.1	0.0	0.0	0.0	1.0	0.0	0.0
**M2/M1**	3.0	1.8	0.0	2.0	16.7	2.1	27.6	12.5	100.0	23.3	50.0	2.4	0.0	3.8	0.0	0.0	2.3	0.0	2.1	0.0	0.9	0.0	0.0	1.0	2.9
**IFN**	0.0	0.0	0.0	0.0	8.3	0.0	13.8	6.2	5.6	100.0	16.7	0.0	0.0	0.0	0.0	0.0	0.0	0.0	0.0	0.0	0.0	0.0	0.0	0.0	0.0
**IL-8**	0.0	0.0	0.0	2.0	8.3	0.0	3.4	2.1	2.4	3.3	100.0	0.0	0.0	0.0	0.0	0.0	0.0	0.0	0.0	0.0	0.0	0.0	0.0	0.0	0.0
**Adipocytes**	0.0	1.8	0.0	0.0	0.0	0.0	3.4	2.1	1.6	0.0	0.0	100.0	7.7	0.0	0.0	0.0	0.0	2.8	0.0	1.2	0.0	0.0	0.0	0.0	0.0
**Glycolysis**	0.0	0.0	0.0	0.0	0.0	0.0	0.0	0.0	0.0	0.0	0.0	1.2	100.0	0.0	0.0	0.0	0.0	5.6	0.0	0.0	0.0	0.0	0.0	1.0	0.0
**IRGS**	0.0	0.0	0.0	0.0	0.0	0.0	0.0	0.0	1.6	0.0	0.0	0.0	0.0	100.0	0.0	0.0	0.0	0.0	0.0	0.0	3.6	0.0	0.0	2.0	0.6
**CIN**	0.0	0.0	2.1	0.0	0.0	0.0	0.0	0.0	0.0	0.0	0.0	0.0	0.0	0.0	100.0	0.0	0.0	0.0	32.6	0.0	0.0	17.9	29.1	5.0	8.1
**ERBB2**	0.0	0.0	0.0	0.0	0.0	0.0	0.0	0.0	0.0	0.0	0.0	0.0	0.0	0.0	0.0	100.0	0.0	0.0	0.0	0.0	1.8	0.0	0.0	2.0	1.7
**HOXA**	4.5	3.5	0.0	0.0	0.0	0.0	0.0	0.0	0.8	0.0	0.0	0.0	0.0	0.0	0.0	0.0	100.0	0.0	0.0	1.2	1.8	0.0	0.0	4.0	2.9
**MITO/ OXPHOS**	0.0	0.0	0.0	0.0	0.0	2.1	0.0	0.0	0.0	0.0	0.0	1.2	15.4	0.0	0.0	0.0	0.0	100.0	1.1	0.0	0.0	0.0	1.0	0.0	0.0
**Proliferation**	1.5	0.0	0.0	0.0	0.0	0.0	0.0	2.1	1.6	0.0	0.0	0.0	0.0	0.0	46.3	0.0	0.0	2.8	100.0	0.0	0.0	12.8	57.3	26.0	17.3
**Reactive stroma**	1.5	0.0	0.0	0.0	0.0	4.3	0.0	0.0	0.0	0.0	0.0	1.2	0.0	0.0	0.0	0.0	2.3	0.0	0.0	100.0	1.8	2.6	1.0	0.0	1.7
**VEGF**	3.0	0.0	0.0	2.0	0.0	0.0	0.0	0.0	0.8	0.0	0.0	0.0	0.0	7.5	0.0	9.5	4.7	0.0	0.0	2.4	100.0	5.1	0.0	4.0	4.0
**70-GES**	0.0	0.0	0.0	0.0	0.0	0.0	0.0	0.0	0.0	0.0	0.0	0.0	0.0	0.0	10.4	0.0	0.0	0.0	5.3	1.2	1.8	100.0	6.8	3.0	1.2
**GGI**	0.0	0.0	0.0	0.0	0.0	2.1	0.0	2.1	0.0	0.0	0.0	0.0	0.0	0.0	44.8	0.0	0.0	2.8	62.1	1.2	0.0	17.9	100.0	17.0	14.5
**RS**	21.2	1.8	0.0	0.0	0.0	0.0	0.0	0.0	0.8	0.0	0.0	0.0	7.7	3.8	7.5	9.5	9.3	0.0	27.4	0.0	3.6	7.7	16.5	100.0	28.3
**PAM50**	28.8	5.3	35.4	0.0	0.0	0.0	0.0	0.0	4.0	0.0	0.0	0.0	0.0	1.9	20.9	14.3	11.6	0.0	31.6	3.5	6.4	5.1	24.3	49.0	100.0
