# Supplementary material for: Gene-expression signature functional annotation of breast cancer tumours in function of age
Source: BMC Med Genomics. 2015 Nov 23;8:80. doi: 10.1186/s12920-015-0153-6 (PMC4657228; doi:10.1186/s12920-015-0153-6)
Supplement: Additional file 9: — Percentage of GO enrichment terms (biological tree) in common between GES’s gene lists, sorted in decreasing order. (PDF 99 kb) [file 12920_2015_153_MOESM9_ESM.pdf]

Additional file 9: Percentage of GO enrichment terms (biological tree) in common between GES's gene lists, sorted in decreasing order.

ER		Molecular-apocrine		Basal-like		Claudin-CD24		B-cell		T-cell		MHC-1		MHC-2		M2/M1		IFN	
%	GES	%	GES	%	GES	%	GES	%	GES	%	GES	%	GES	%	GES	%	GES	%	GES
28.8	PAM50	17.5	ER	35.4	PAM50	2.0	Molecular-apocrine	25.0	MHC-1	8.5	MHC-2	27.6	M2/M1	12.5	M2/M1	6.3	MHC-1	23.3	M2/M1
21.2	RS	5.3	PAM50	2.1	Claudin-CD24	2.0	Basal-like	16.7	M2/M1	4.3	Reactive stroma	17.2	MHC-2	10.4	MHC-1	5.6	IFN	13.3	MHC-1
15.2	Molecular-apocrine	3.5	HOXA	2.1	CIN	2.0	T-cell	8.3	T-cell	2.1	Molecular-apocrine	13.8	IFN	8.3	T-cell	4.8	MHC-2	10.0	MHC-2
4.5	HOXA	1.8	Claudin-CD24	0.0	ER	2.0	MHC-1	8.3	MHC-2	2.1	Claudin-CD24	10.3	B-cell	6.2	IFN	4.0	PAM50	3.3	B-cell
3.0	M2/M1	1.8	T-cell	0.0	Molecular-apocrine	2.0	M2/M1	8.3	IFN	2.1	B-cell	3.4	Claudin-CD24	2.1	ER	2.4	IL-8	3.3	IL-8
3.0	VEGF	1.8	M2/M1	0.0	B-cell	2.0	IL-8	8.3	IL-8	2.1	MHC-1	3.4	T-cell	2.1	B-cell	1.6	ER	0.0	ER
1.5	MHC-2	1.8	Adipocytes	0.0	T-cell	2.0	VEGF	0.0	ER	2.1	M2/M1	3.4	IL-8	2.1	IL-8	1.6	B-cell	0.0	Molecular-apocrine
1.5	Proliferation	1.8	RS	0.0	MHC-1	0.0	ER	0.0	Molecular-apocrine	2.1	MITO/OXPHOS	3.4	Adipocytes	2.1	Adipocytes	1.6	Adipocytes	0.0	Basal-like
1.5	Reactive stroma	0.0	Basal-like	0.0	MHC-2	0.0	B-cell	0.0	Basal-like	2.1	GGI	0.0	ER	2.1	Proliferation	1.6	IRGS	0.0	Claudin-CD24
0.0	Basal-like	0.0	B-cell	0.0	M2/M1	0.0	MHC-2	0.0	Claudin-CD24	0.0	ER	0.0	Molecular-apocrine	2.1	GGI	1.6	Proliferation	0.0	T-cell
0.0	Claudin-CD24	0.0	MHC-1	0.0	IFN	0.0	IFN	0.0	Adipocytes	0.0	Basal-like	0.0	Basal-like	0.0	Molecular-apocrine	0.8	Molecular-apocrine	0.0	Adipocytes
0.0	B-cell	0.0	MHC-2	0.0	IL-8	0.0	Adipocytes	0.0	Glycolysis	0.0	IFN	0.0	Glycolysis	0.0	Basal-like	0.8	Claudin-CD24	0.0	Glycolysis
0.0	T-cell	0.0	IFN	0.0	Adipocytes	0.0	Glycolysis	0.0	IRGS	0.0	IL-8	0.0	IRGS	0.0	Claudin-CD24	0.8	T-cell	0.0	IRGS
0.0	MHC-1	0.0	IL-8	0.0	Glycolysis	0.0	IRGS	0.0	CIN	0.0	Adipocytes	0.0	CIN	0.0	Glycolysis	0.8	HOXA	0.0	CIN
0.0	IFN	0.0	Glycolysis	0.0	IRGS	0.0	CIN	0.0	ERBB2	0.0	Glycolysis	0.0	ERBB2	0.0	IRGS	0.8	VEGF	0.0	ERBB2
0.0	IL-8	0.0	IRGS	0.0	ERBB2	0.0	ERBB2	0.0	HOXA	0.0	IRGS	0.0	HOXA	0.0	CIN	0.8	RS	0.0	HOXA
0.0	Adipocytes	0.0	CIN	0.0	HOXA	0.0	HOXA	0.0	MITO/OXPHOS	0.0	CIN	0.0	MITO/OXPHOS	0.0	ERBB2	0.0	Basal-like	0.0	MITO/OXPHOS
0.0	Glycolysis	0.0	ERBB2	0.0	MITO/OXPHOS	0.0	MITO/OXPHOS	0.0	Proliferation	0.0	ERBB2	0.0	Proliferation	0.0	HOXA	0.0	Glycolysis	0.0	Proliferation
0.0	IRGS	0.0	MITO/OXPHOS	0.0	Proliferation	0.0	Proliferation	0.0	Reactive stroma	0.0	HOXA	0.0	Reactive stroma	0.0	MITO/OXPHOS	0.0	CIN	0.0	Reactive stroma
0.0	CIN	0.0	Proliferation	0.0	Reactive stroma	0.0	Reactive stroma	0.0	VEGF	0.0	Proliferation	0.0	VEGF	0.0	Reactive stroma	0.0	ERBB2	0.0	VEGF
0.0	ERBB2	0.0	Reactive stroma	0.0	VEGF	0.0	70-GES	0.0	70-GES	0.0	VEGF	0.0	70-GES	0.0	VEGF	0.0	MITO/OXPHOS	0.0	70-GES
0.0	MITO/OXPHOS	0.0	VEGF	0.0	70-GES	0.0	GGI	0.0	GGI	0.0	70-GES	0.0	GGI	0.0	70-GES	0.0	Reactive stroma	0.0	GGI
0.0	70-GES	0.0	70-GES	0.0	GGI	0.0	RS	0.0	RS	0.0	RS	0.0	RS	0.0	RS	0.0	70-GES	0.0	RS
0.0	GGI	0.0	GGI	0.0	RS	0.0	PAM50	0.0	PAM50	0.0	PAM50	0.0	PAM50	0.0	PAM50	0.0	GGI	0.0	PAM50

IL-8		Adipocytes		Glycolysis		IRGS		CIN		ERBB2		HOXA		MITO/OXPHOS		Proliferation		Reactive stroma	
%	GES	%	GES	%	GES	%	GES	%	GES	%	GES	%	GES	%	GES	%	GES	%	GES
50.0	M2/M1	2.4	M2/M1	15.4	MITO/OXPHOS	7.5	VEGF	46.3	Proliferation	14.3	PAM50	11.6	PAM50	5.6	Glycolysis	62.1	GGI	3.5	PAM50
16.7	Claudin-CD24	1.2	Molecular-apocrine	7.7	Adipocytes	3.8	M2/M1	44.8	GGI	9.5	VEGF	9.3	RS	2.8	T-cell	32.6	CIN	2.4	T-cell
16.7	B-cell	1.2	MHC-1	7.7	RS	3.8	RS	20.9	PAM50	9.5	RS	7.0	ER	2.8	Adipocytes	31.6	PAM50	2.4	VEGF
16.7	MHC-1	1.2	MHC-2	0.0	ER	1.9	PAM50	10.4	70-GES	0.0	ER	4.7	Molecular-apocrine	2.8	Proliferation	27.4	RS	1.2	ER
16.7	MHC-2	1.2	Glycolysis	0.0	Molecular-apocrine	0.0	ER	7.5	RS	0.0	Molecular-apocrine	4.7	VEGF	2.8	GGI	5.3	70-GES	1.2	Adipocytes
16.7	IFN	1.2	MITO/OXPHOS	0.0	Basal-like	0.0	Molecular-apocrine	1.5	Basal-like	0.0	Basal-like	2.3	M2/M1	0.0	ER	2.1	M2/M1	1.2	HOXA
0.0	ER	1.2	Reactive stroma	0.0	Claudin-CD24	0.0	Basal-like	0.0	ER	0.0	Claudin-CD24	2.3	Reactive stroma	0.0	Molecular-apocrine	1.1	ER	1.2	70-GES
0.0	Molecular-apocrine	0.0	ER	0.0	B-cell	0.0	Claudin-CD24	0.0	Molecular-apocrine	0.0	B-cell	0.0	Basal-like	0.0	Basal-like	1.1	MHC-2	1.2	GGI
0.0	Basal-like	0.0	Basal-like	0.0	T-cell	0.0	B-cell	0.0	Claudin-CD24	0.0	T-cell	0.0	Claudin-CD24	0.0	Claudin-CD24	1.1	MITO/OXPHOS	0.0	Molecular-apocrine
0.0	T-cell	0.0	Claudin-CD24	0.0	MHC-1	0.0	T-cell	0.0	B-cell	0.0	MHC-1	0.0	B-cell	0.0	B-cell	0.0	Molecular-apocrine	0.0	Basal-like
0.0	Adipocytes	0.0	B-cell	0.0	MHC-2	0.0	MHC-1	0.0	T-cell	0.0	MHC-2	0.0	T-cell	0.0	MHC-1	0.0	Basal-like	0.0	Claudin-CD24
0.0	Glycolysis	0.0	T-cell	0.0	M2/M1	0.0	MHC-2	0.0	MHC-1	0.0	M2/M1	0.0	MHC-1	0.0	MHC-2	0.0	Claudin-CD24	0.0	B-cell
0.0	IRGS	0.0	IFN	0.0	IFN	0.0	IFN	0.0	MHC-2	0.0	IFN	0.0	MHC-2	0.0	M2/M1	0.0	B-cell	0.0	MHC-1
0.0	CIN	0.0	IL-8	0.0	IL-8	0.0	IL-8	0.0	M2/M1	0.0	IL-8	0.0	IFN	0.0	IFN	0.0	T-cell	0.0	MHC-2
0.0	ERBB2	0.0	IRGS	0.0	IRGS	0.0	Adipocytes	0.0	IFN	0.0	Adipocytes	0.0	IL-8	0.0	IL-8	0.0	MHC-1	0.0	M2/M1
0.0	HOXA	0.0	CIN	0.0	CIN	0.0	Glycolysis	0.0	IL-8	0.0	Glycolysis	0.0	Adipocytes	0.0	IRGS	0.0	IFN	0.0	IFN
0.0	MITO/OXPHOS	0.0	ERBB2	0.0	ERBB2	0.0	CIN	0.0	Adipocytes	0.0	IRGS	0.0	Glycolysis	0.0	CIN	0.0	IL-8	0.0	IL-8
0.0	Proliferation	0.0	HOXA	0.0	HOXA	0.0	ERBB2	0.0	Glycolysis	0.0	CIN	0.0	IRGS	0.0	ERBB2	0.0	Adipocytes	0.0	Glycolysis
0.0	Reactive stroma	0.0	Proliferation	0.0	Proliferation	0.0	HOXA	0.0	IRGS	0.0	HOXA	0.0	CIN	0.0	HOXA	0.0	Glycolysis	0.0	IRGS
0.0	VEGF	0.0	VEGF	0.0	Reactive stroma	0.0	MITO/OXPHOS	0.0	ERBB2	0.0	MITO/OXPHOS	0.0	ERBB2	0.0	Reactive stroma	0.0	IRGS	0.0	CIN
0.0	70-GES	0.0	70-GES	0.0	VEGF	0.0	Proliferation	0.0	HOXA	0.0	Proliferation	0.0	MITO/OXPHOS	0.0	VEGF	0.0	ERBB2	0.0	ERBB2
0.0	GGI	0.0	GGI	0.0	70-GES	0.0	Reactive stroma	0.0	MITO/OXPHOS	0.0	Reactive stroma	0.0	Proliferation	0.0	70-GES	0.0	HOXA	0.0	MITO/OXPHOS
0.0	RS	0.0	RS	0.0	GGI	0.0	70-GES	0.0	Reactive stroma	0.0	70-GES	0.0	70-GES	0.0	RS	0.0	Reactive stroma	0.0	Proliferation
0.0	PAM50	0.0	PAM50	0.0	PAM50	0.0	GGI	0.0	VEGF	0.0	GGI	0.0	GGI	0.0	PAM50	0.0	VEGF	0.0	RS

VEGF		70-GES		GGI		RS		PAM50	
%	GES	%	GES	%	GES	%	GES	%	GES
6.4	PAM50	17.9	CIN	57.3	Proliferation	49.0	PAM50	28.3	RS
3.6	IRGS	17.9	GGI	29.1	CIN	26.0	Proliferation	17.3	Proliferation
3.6	RS	12.8	Proliferation	24.3	PAM50	17.0	GGI	14.5	GGI
1.8	ER	7.7	RS	16.5	RS	14.0	ER	11	ER
1.8	ERBB2	5.1	VEGF	6.8	70-GES	5.0	CIN	9.8	Basal-like
1.8	HOXA	5.1	PAM50	1.0	T-cell	4.0	HOXA	8.1	CIN
1.8	Reactive stroma	2.6	Reactive stroma	1.0	MHC-2	4.0	VEGF	4.0	VEGF
1.8	70-GES	0.0	ER	1.0	MITO/OXPHOS	3.0	70-GES	2.9	M2/M1
0.9	Claudin-CD24	0.0	Molecular-apocrine	1.0	Reactive stroma	2.0	IRGS	2.9	HOXA
0.9	M2/M1	0.0	Basal-like	0.0	ER	2.0	ERBB2	1.7	Molecular-apocrine
0.0	Molecular-apocrine	0.0	Claudin-CD24	0.0	Molecular-apocrine	1.0	Molecular-apocrine	1.7	ERBB2
0.0	Basal-like	0.0	B-cell	0.0	Basal-like	1.0	M2/M1	1.7	Reactive stroma
0.0	B-cell	0.0	T-cell	0.0	Claudin-CD24	1.0	Glycolysis	1.2	70-GES
0.0	T-cell	0.0	MHC-1	0.0	B-cell	0.0	Basal-like	0.6	IRGS
0.0	MHC-1	0.0	MHC-2	0.0	MHC-1	0.0	Claudin-CD24	0.0	Claudin-CD24
0.0	MHC-2	0.0	M2/M1	0.0	M2/M1	0.0	B-cell	0.0	B-cell
0.0	IFN	0.0	IFN	0.0	IFN	0.0	T-cell	0.0	T-cell
0.0	IL-8	0.0	IL-8	0.0	IL-8	0.0	MHC-1	0.0	MHC-1
0.0	Adipocytes	0.0	Adipocytes	0.0	Adipocytes	0.0	MHC-2	0.0	MHC-2
0.0	Glycolysis	0.0	Glycolysis	0.0	Glycolysis	0.0	IFN	0.0	IFN
0.0	CIN	0.0	IRGS	0.0	IRGS	0.0	IL-8	0.0	IL-8
0.0	MITO/OXPHOS	0.0	ERBB2	0.0	ERBB2	0.0	Adipocytes	0.0	Adipocytes
0.0	Proliferation	0.0	HOXA	0.0	HOXA	0.0	MITO/OXPHOS	0.0	Glycolysis
0.0	GGI	0.0	MITO/OXPHOS	0.0	VEGF	0.0	Reactive stroma	0.0	MITO/OXPHOS
